# Comparison of mutations in human parainfluenza viruses during passage in primary human bronchial/tracheal epithelial air-liquid interface cultures and cell lines

**DOI:** 10.1128/spectrum.01164-24

**Published:** 2024-07-30

**Authors:** Satoko Sugimoto, Miyuki Kawase, Reiko Suwa, Yohei Kume, Mina Chishiki, Takashi Ono, Hisao Okabe, Sakurako Norito, Ken-Ichi Hanaki, Mitsuaki Hosoya, Koichi Hashimoto, Kazuya Shirato

**Affiliations:** 1 Department of Virology III, National Institute of Infectious Diseases, Tokyo, Japan; 2 Research Center for Biosafety, Laboratory Animal, and Pathogen Bank, National Institute of Infectious Diseases, Tokyo, Japan; 3 Department of Pediatrics, School of Medicine, Fukushima Medical University, Fukushima, Japan; Wright State University, Dayton, Ohio, USA

**Keywords:** air-liquid interface culture, cell culture adaptation, hemagglutinin neuraminidase protein, human parainfluenza virus, quasispecies

## Abstract

Human parainfluenza virus (HPIV) causes respiratory infections, which are exacerbated in children and older people. Correct evaluation of viral characteristics is essential for the study of countermeasures. However, adaptation of viruses to cultured cells during isolation or propagation might select laboratory passage-associated mutations that modify the characteristics of the virus. It was previously reported that adaptation of HPIV3, but not other HPIVs, was avoided in human airway epithelia. To examine the influence of laboratory passage on the genomes of HPIV1–HPIV4, we evaluated the occurrence of mutations after passage in primary human bronchial/tracheal epithelial cell air-liquid interface (HBTEC-ALI) culture and conventional cultured cells (Vero cells expressing the transmembrane protease, serine 2, and normal Vero cells). The occurrence of mutations was significantly lower in HBTEC-ALI than in conventional culture. In HBTEC-ALI culture, most of the mutations were silent or remained at low variant frequency, resulting in less impact on the viral consensus sequence. In contrast, passage in conventional culture induced or selected genetic mutations at high frequency with passage-associated unique substitutions. High mutagenesis of hemagglutinin-neuraminidase was commonly observed in all four HPIVs, and mutations even occurred in a single passage. In addition, in HPIV1 and HPIV2, mutations in the large protein were more frequent. These results indicate that passage in HBTEC-ALI culture is more suitable than conventional culture for maintaining the original characteristics of clinical isolates in all four HPIVs, which can help with the understanding of viral pathogenesis.

**IMPORTANCE:**

Adaptation of viruses to cultured cells can increase the risk of misinterpretation in virological characterization of clinical isolates. In human parainfluenza virus (HPIV) 3, it has been reported that the human airway epithelial and lung organoid models are preferable for the study of viral characteristics of clinical strains without mutations. Therefore, we analyzed clinical isolates of all four HPIVs for the occurrence of mutations after five laboratory passages in human bronchial/tracheal epithelial cell air-liquid interface (HBTEC-ALI) or conventional culture. We found a high risk of hemagglutinin-neuraminidase mutagenesis in all four HPIVs in conventional cultured cells. In addition, in HPIV1 and HPIV2, mutations of the large protein were also more frequent in conventional cultured cells than in HBTEC-ALI culture. HBTEC-ALI culture was useful for maintaining the original sequence and characteristics of clinical isolates in all four HPIVs. The present study contributes to the understanding of HPIV pathogenesis and antiviral strategies.

## INTRODUCTION

Human parainfluenza virus (HPIV) is an enveloped, negative-strand RNA virus belonging to the family *Paramyxoviridae*. Four species are pathogenic for humans: *Respirovirus laryngotracheitidis* (HPIV1), *Orthorubulavirus laryngotracheitidis* (HPIV2), *Respirovirus pneumoniae* (HPIV3), and *Orthorubulavirus hominis* (HPIV4). They have some similarities, such as the organization of the viral genome (3′-N-P-M-F-HN-L-5′), neuraminidase activity, agglutination of red blood cells, and causing respiratory infections (1). HPIVs have been isolated and propagated in primary cultures of monkey kidney epithelial, LLC-MK2, Vero, CV-I, Wish, HMV-II, HEp-2, MDCK, BHK, HeLa, primary human embryo, KB, Am, HEB′ L929, HEF (1), MNT-1 (2), VeroE6 (3), and PLC/PRF/5 (4) cells, often with trypsin.

However, it has been demonstrated that growth of HPIV3 in cultured cells alters the dynamics of hemagglutinin–neuraminidase (HN) and fusion (F) protein and the viral characteristics (5–9). Also, laboratory strains isolated or passaged in cultured cells do not maintain the same characteristics as community circulating viruses (5, 7). Therefore, in HPIV3, human airway epithelial and lung organoid models are suitable for the study of viral growth and function of clinical strains, without the selective pressure that is present in conventional monolayer culture (7, 8, 10). However, this has not yet been studied fully in other HPIVs. Normal human bronchial/tracheal epithelial cell air-liquid interface (HBTEC-ALI) culture is one of the human airway epithelial models. It can mimic the physiological environment of the airway epithelium, such as ciliary movement and mucus production, and successfully propagate many species of respiratory viruses, including the four HPIVs (11). This suggests that the human airway epithelial model is also useful for culture of clinical isolates of various viruses. However, it would be better if a virus without mutations could be obtained in conventional cultured cells to reduce costs.

Adaptation to cultured cells is observed for many viruses, and even a single passage in cultured cells has the potential to cause mutations (5, 12). Passaged viruses exhibit different characteristics from the original viruses (5, 13), drug sensitivity (4, 14), and pathogenicity in laboratory animals (15). Passage-selected mutations include the multibasic cleavage site in the spike protein in severe acute respiratory syndrome coronavirus 2 (SARS-CoV-2) (14) and hemagglutinin (HA) protein in influenza A virus (IAV) (16). These have been reported to be less frequent in cultured cells expressing host factors involved in infection, such as proteases (14) and sialic acids (16). This suggests that mimicking the natural infection environment is important for minimizing changes in the viral genome caused by passage. Viruses belonging to the family *Paramyxoviridae*, including HPIVs, require F protein activation by proteolytic cleavage. Some viruses are cleaved by the ubiquitous furin, tissue-specific proteases, or extracellular proteases (17). Thus, when the virus is propagated in cultured cells lacking necessary proteases, exogenous proteases need to be added. Our group previously reported that HPIVs proliferate well without trypsin treatment in Vero cells expressing the transmembrane protease, serine 2 (Vero/TMPRSS2), one of the type II transmembrane serine proteases expressed in human airway epithelia *in vivo* (18).

Therefore, the present study was conducted to determine (i) whether laboratory passage in HBTEC-ALI culture has less influence on the genomes of HPIV1, HPIV2, and HPIV4 compared with conventional cell cultures, similar to HPIV3, and (ii) whether Vero/TMPRSS2 cells are also useful for HPIV passage, similar to coronaviruses. We assessed by next-generation sequencing (NGS) the occurrence and characteristics of mutations of clinical isolates of HPIV1–HPIV4 before and after five blind passages in HBTEC-ALI, Vero/TMPRSS2, and Vero cell culture.

## RESULTS

### Effect of HPIV passages in HBTEC-ALI and conventional cell cultures

All 14 HPIV clinical isolates replicated in HBTEC-ALI and Vero/TMPRSS2 cell culture. In contrast, HPIV1 (*n* = 4), HPIV3 (*n* = 3), and HPIV4 (*n* = 3) isolates were not able to replicate in Vero cells without trypsin, although HPIV2 (*n* = 4) isolates were able. Changes in the cytopathic effects (CPEs) (Table 1) and genome copy number (Table S1) through blind passages were examined. No obvious CPE was observed in HBTEC-ALI culture, whereas infected Vero/TMPRSS2 and Vero cells showed rounding, detachment, and/or syncytial formation. All four HPIV2 isolates showed syncytial formation in Vero/TMPRSS2 at first passage (P1). In HPIV1, HPIV3, and HPIV4, syncytial formation was not a common observation in Vero/TMPRSS2 cells at P1, except for PIV3_Fukushima_O726_2019 and PIV4b_Fukushima_OR476_2022, but became more apparent over passage. After five passages (P5), syncytial formation was observed in 11 of 14 isolates, except for PIV1_Fukushima_O77_2018, PIV1_Fukushima_H171_2018, and PIV3_Fukushima_O330_2018.

**TABLE 1:** CPE observations at each passage

Culture (passage)	Species	Name	Observations of CPE				
			P1	P2	P3	P4	P5
Vero/TMPRSS2	*Respirovirus laryngotracheitidis* (human parainfluenza virus 1)	PIV1_Fukushima_O77_2018	−	−	+	+	+^*a*^
		PIV1_Fukushima_H171_2018	−	−	−	+	+^*a*^
		PIV1_Fukushima_O425_2018	−	−	−	+	+
		PIV1_Fukushima_O503_2019	−	+	+	+	+
	*Orthorubulavirus laryngotracheitidis* (human parainfluenza virus 2)	PIV2_Fukushima_O13_2018	+	+	+	+	+
		PIV2_Fukushima_O33_2018	+	+	+	+	+
		PIV2_Fukushima_O836_2019	+	+	+	+	+
		PIV2_Fukushima_O861_2019	+	+	+	+	+
	*Respirovirus pneumoniae* (human parainfluenza virus 3)	PIV3_Fukushima_O330_2018	−	+	+	+	+^*a*^
		PIV3_Fukushima_O644_2019	−	+	+	+	+
		PIV3_Fukushima_O726_2019	+	+	+	+	+
	*Orthorubulavirus hominis* (human parainfluenza virus 4)	PIV4a_Fukushima_H725_2019	−	−	−	+	+
		PIV4b_Fukushima_O896_2019	−	−	+	+	+
		PIV4b_Fukushima_OR476_2022	+	+	+	+	+
Vero	*Orthorubulavirus laryngotracheitidis* (human parainfluenza virus 2)	PIV2_Fukushima_O13_2018	−	−	+	−	+
		PIV2_Fukushima_O33_2018	−	−	−	+	+
		PIV2_Fukushima_O836_2019	−	−	+	−	+
		PIV2_Fukushima_O861_2019	+	+	+	+	+

^*a*^ No syncytia were observed at P5.

**Fig 1:**
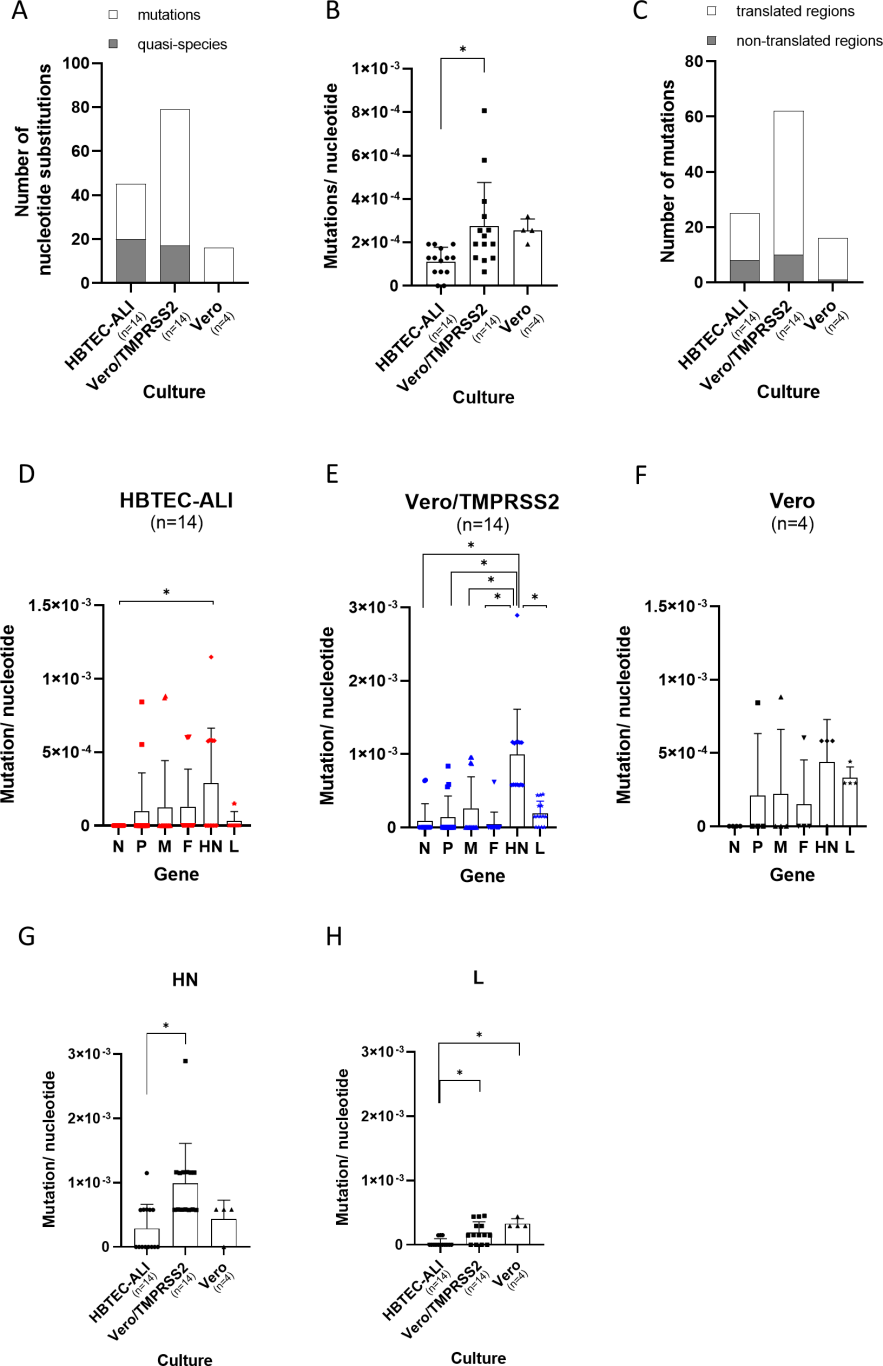
Mutation rate when HPIVs were passaged in each culture. (A) Total number of nucleotide substitutions with ≥10% frequency in P5 sequences are shown. Mutations in P5 viruses were defined as those in which quasispecies were excluded from the nucleotide substitutions. The quasispecies were defined as variants that were present at ≥1% frequency in the P0 inoculum. (B) Mutation rate of each isolate after five blind passages in different cultures. (C) Regions of mutations observed in P5 sequences. (D to F) Mutation rate of each gene after five blind passages in (D) HBTEC-ALI, (E) Vero/TMPRSS2, and (F) Vero cells. (G and H) Mutation rate of (G) *HN* and (H) *L* gene in each culture. “n” indicates the number of isolates used in the study. Statistics were calculated using Tukey’s multiple comparisons test (*P < 0.05).

After five passages in each culture, P1, P3, and P5 sequences were compared with the original sequences (P0), and nucleotide substitutions with >10% frequency were detected by variant analysis (Tables S2 through S4). The mutations in P1, P3, and P5 sequences were defined as nucleotide substitutions, except for the quasispecies that originally existed in the P0 inoculum at >1% frequency (Table S5). Nearly half of nucleotide substitutions observed in P5 sequences were quasispecies in HBTEC-ALI culture (Fig. 1A). In contrast, mutations were abundant in conventional cultured cells (Fig. 1A). Some of the mutations that occurred at P1 and P3 disappeared or were maintained through to P5, while some increased (Fig. S1).

The mutation rate of genomic nucleotide sequences after P5 was significantly lower in HBTEC-ALI than in Vero/TMPRSS2 cell culture (Fig. 1B). The coding region of the HPIV genome was more stable in HBTEC-ALI than in conventional cell culture (Fig. 1C). Contrary to our expectations, the mutation rate was similar when HPIV was passaged in Vero and Vero/TMPRSS2 cells (Fig. 1B). The mutation rate of the nucleotide sequence in each gene was also determined, and mutations were more likely to have occurred in *HN* than in other genes in Vero/TMPRSS2 cells (Fig. 1D through F). The *HN* gene had significantly higher mutation rates in Vero/TMPRSS2 cells than in HBTEC-ALI culture (Fig. 1G). The *L* gene also had significantly higher mutation rates in Vero/TMPRSS2 and Vero cells than in HBTEC-ALI culture, although not as high as for *HN* (Fig. 1H). In other genes, no significant differences were observed between different culture methods (Fig. S2).

### Conservation of quasispecies in HBTEC-ALI culture

**Fig 2:**
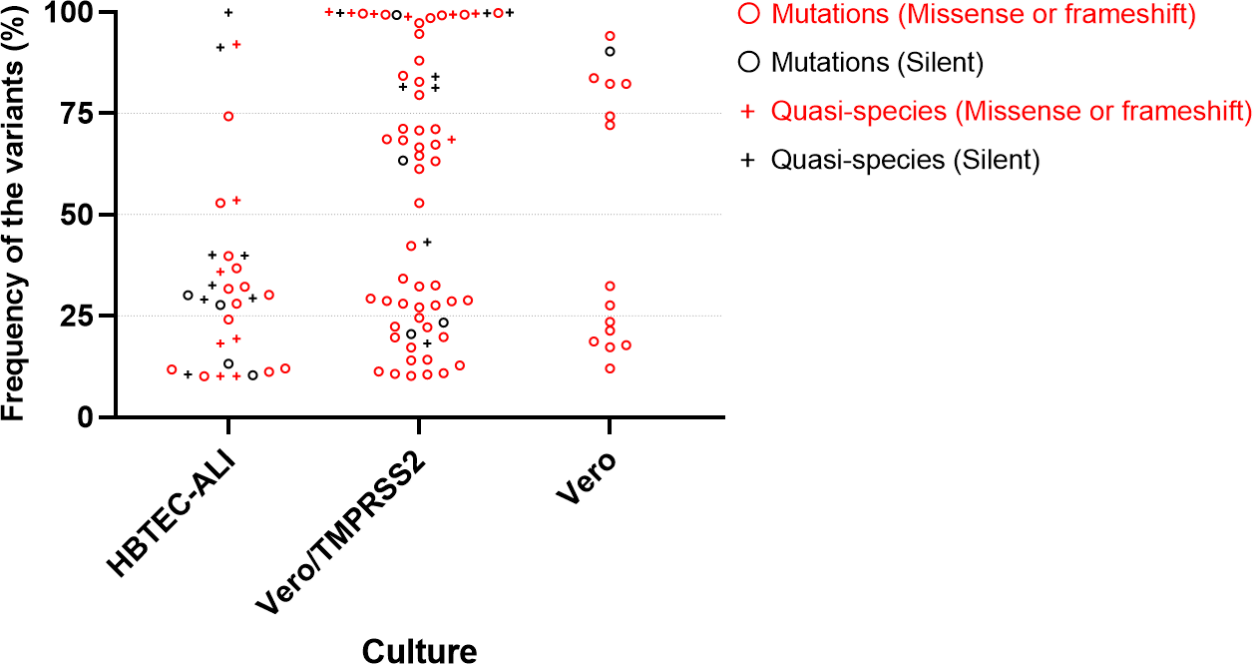
Variant frequency of all nucleotide substitutions that occurred in the translated region after five blind passages in HBTEC-ALI, Vero/TMPRSS2, and Vero cells. Circle (○) shows newly occurring mutations after five blind passages, and cross (+) shows quasispecies that existed in the P0 inoculum. Black color shows mutations with no amino acid alteration (silent) and red color mutations with amino acid alterations (missense or frameshift).

To assess the effect of nucleotide substitutions on the characteristics of HPIVs, we analyzed whether these mutations/quasispecies in the coding regions of P5 sequences led to amino acid substitutions. Silent substitutions were more often seen in HBTEC-ALI (12/32, 38%) compared with Vero/TMPRSS2 (12/67, 18%) and Vero cell (1/15, 7%) culture (Fig. 2, black). The frequency of the variants in Fig. 2 represented the fraction of variant sequencing reads within a reference P0 genomic locus. There were 20 missense mutations/quasispecies in HBTEC-ALI culture (Fig. 2, red). Sixteen (80%) of these had <50% frequency, which indicated no apparent amino acid substitutions. In contrast, more than half the missense mutations/quasispecies in Vero/TMPRSS2 cells (30/55, 55%) showed >50% frequency, which indicated that passage resulted in amino acid substitutions. In Vero cells, only mutations were seen and 6 of 15 (40%) showed >50% frequency, that is, passage resulted in six apparent amino acid substitutions. These results indicate that HBTEC-ALI culture mostly maintained quasispecies and silent substitutions at low variant frequency and had less impact on the whole viral sequence. In contrast, passage in conventional cultured cells could cause newly developed mutations that were often accompanied with amino acid alterations and reached high variant frequency sufficient to replace the P5 sequences (Fig. 2).

### Amino acid mutations in HN and large (L) protein after passage

**Fig 3:**
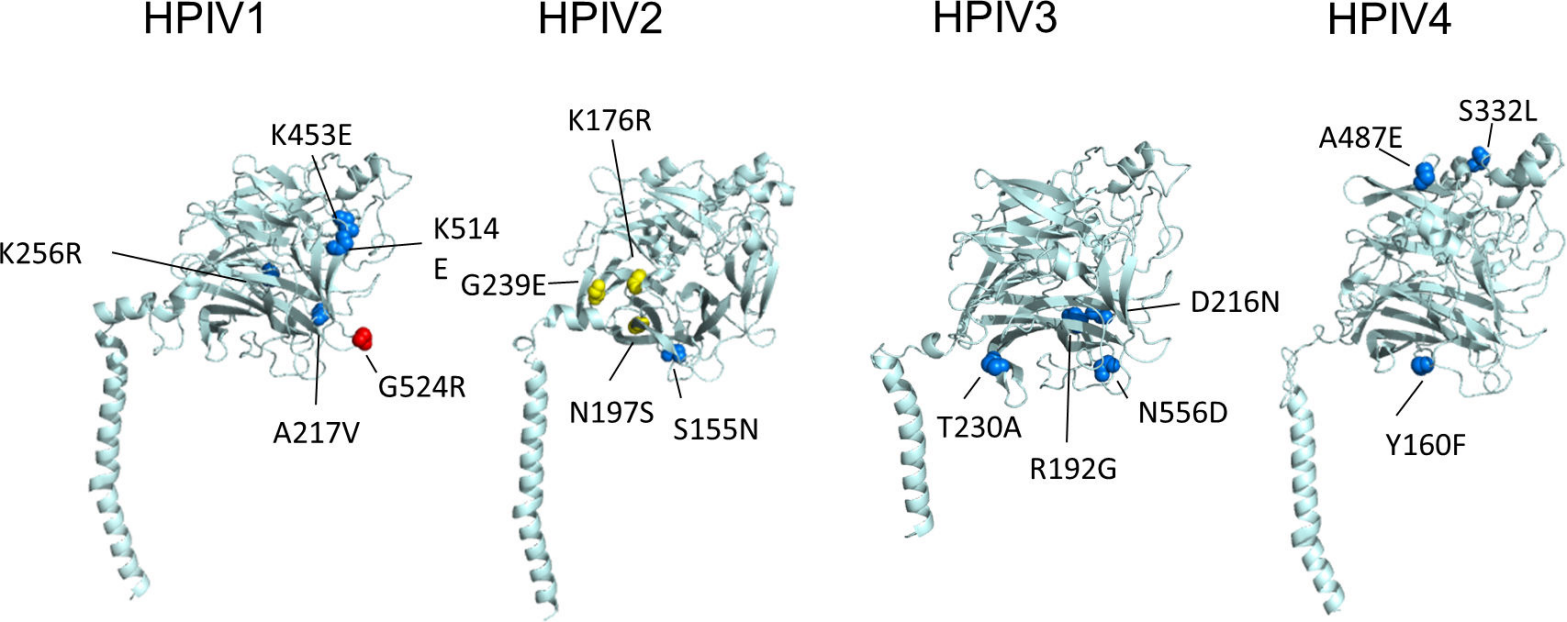
Amino acid mutations occurring in HN protein. The cartoon representation shows the position of amino acid residues shown in Table 2, highlighting the mutations >50% variant frequency in HBTEC-ALI (red sphere), Vero/TMPRSS2 (blue sphere), and Vero (yellow sphere) cells.

**Fig 4:**
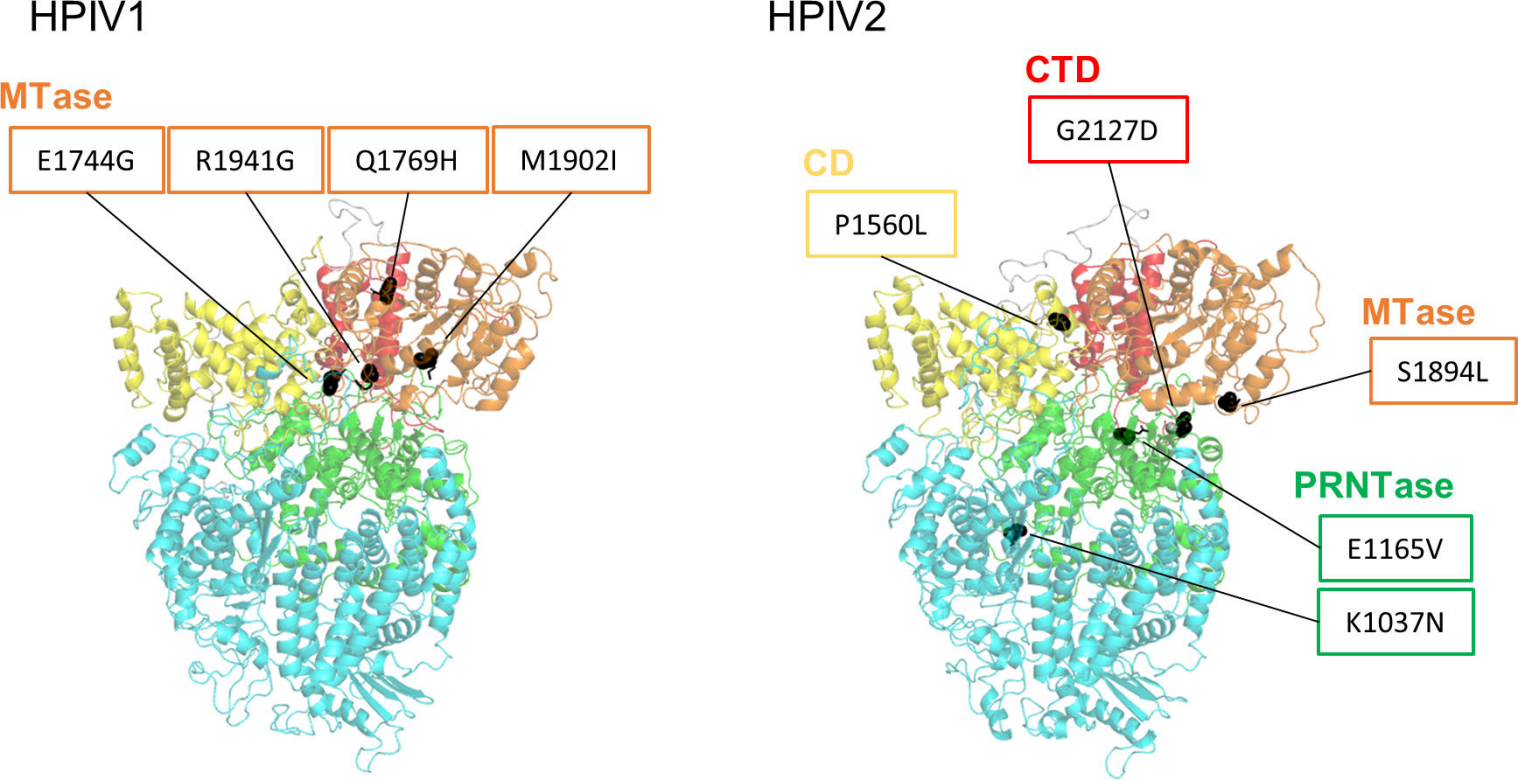
Amino acid mutations occurring in L protein. The cartoon representation shows the position of amino acid residues (black sphere), highlighting the mutations >50% variant frequency shown in Table 3. The domains are described based on previous reports (19): RNA-dependent RNA polymerase (RdRp), cyan; poly-ribonucleotidyltransferase (PRNTase), green; connecting domain (CD), yellow; methyltransferase (MTase), orange; C-terminal domain (CTD), red.

We focused on the amino acid mutations with >50% variant frequency, because they were thought to have the potential to change the consensus sequence and characteristics of the virus. A total of 31 amino acid mutations were identified in P5 with >50% variant frequency. Sixteen amino acid mutations were identified in *HN* (Table 2). In other genes, three were found in *P*, two in *M*, one in *F*, and nine in *L* (Table 3). Notably, the two isolates, PIV3_Fukushima_O726_2019 and PIV4b_Fukushima_OR476_2022, had mutations with high variant frequencies from the time of P1 (Table 2). All the amino acid mutations in HN protein were located in the globular head region (Fig. 3). To examine whether the amino acid mutations in HN protein occurred at inherently variable sites, all registered HN protein sequences were compared. Some amino acid mutations that were not found in the registered HN protein sequences were considered to be unique to the present five passages (Table 1, bold), and passages in conventional cultured cells induced unnatural amino acid mutations. Mutations G524R in HBTEC-ALI and G239E in Vero cell culture were not seen in the registered HN protein sequences, indicating that they were unique to the present five passages. However, the amino acid mutations in such variable amino acid sites were also detected by direct sequencing of clinical specimens (Table 1, asterisks), suggesting that they could have been induced by cell passage and spontaneously. Of the nine amino acid mutations in L protein, none were located in the RNA-dependent RNA polymerase domain (Fig. 4, cyan). In HPIV1, all four mutations were found in methyltransferases domain, whereas in HPIV2, they were located in various domains (Fig. 4).

**TABLE 2:** List of nucleotide and amino acid mutations in HN protein with >50% variant frequency^*e*^^,*f*^

Species	Culture (passage)	Name	Nucleotide	Reference	Allele	Frequency			Amino acid	Reference	Mutation^*a*^	Amino acid variation (number)^*b*^	*n* ^*c*^
						P1	P3	P5					
*Respirovirus* *laryngotracheitidis* (human parainfluenza virus 1)	HBTEC-ALI	PIV1_Fukushima_O503_2019	8449	G	A	-	44.5	74.3	524	G	R	G (491), V (6), E (2)	499
	Vero/TMPRSS2	PIV1_Fukushima_H171_2018	7531	C	T	-	78.3	99.4	**217**	**A**	**V**	**A (479**)	479
			8421	A	G	-	-	94.6	514	K	E	K (472), E (23), R (5)	500
		PIV1_Fukushima_O425_2018	8236	A	G	-	26.4	67.3	453	K	E	K (289), R (244), E (8), Q (1)	542
		PIV1_Fukushima_O503_2019	7646	A	G	-	99.6	97.3	256	K	R	K (479), R (4), N (1), I (1)	485
*Orthorubulavirus laryngotracheitidis* (human parainfluenza virus 2)	Vero/TMPRSS2	PIV2_Fukushima_O836_2019	7274	G	A	-	-	71.2	**155**	**S**	**N**	**S (187**)	187
	Vero	PIV2_Fukushima_O33_2018	7535	G	A	-	-	72.1	239	G	E	G (236), R (1)	237
		PIV2_Fukushima_O836_2019	7337	A	G	-	-	82.3	176	K	R	K (185), R (1), N (1)	187
		PIV2_Fukushima_O861_2019	7404	A	G	-	33.6	94.1	197	N	S	N (206), S (1), D (1)	208
*Respirovirus pneumoniae* (human parainfluenza virus 3)	Vero/TMPRSS2	PIV3_Fukushima_O330_2018	8471	A	G	-	99.4	99.8	556	N	D*^d^	dN (1418), D* (12), I (2), B (1), T (1)	1434
		PIV3_Fukushima_O644_2019	7493	A	G	-	89.5	71.1	230	T	A	T (1814), A (4)	1818
		PIV3_Fukushima_O726_2019	7379	A	G	-	97.0	98.5	192	R	G	R (1612), G (5), K (5), I (3)	1625
			7451	G	A	84.4	98.7	99.2	216	D	N*^d^	D (1741), E (3), N* (2), G (1), B (1)	1748
*Orthorubulavirus hominis* (human parainfluenza virus 4)	Vero/TMPRSS2	PIV4a_Fukushima_H725_2019	8049	A	T	NA	75.4	84.3	**160**	**Y**	**F**	**Y (131**)	131
		PIV4b_Fukushima_O896_2019	8555	C	T	NA	-	61.3	**332**	**S**	**L**	**S (151**)	151
		PIV4b_Fukushima_OR476_2022	9022	C	A	84.9	81.8	79.5	**487**	**A**	**E**	**A (127**)	127

^*a*^ We searched the registered HN protein sequences that had the indicated amino acid, and if it was also included in the direct sequence from a clinical specimen without growth in cultured cells, it is indicated by an asterisk.

^*b*^ Amino acid variations were examined in all registered HN protein sequences, and the number of registered sequences having the amino acid residues is shown.

^*c*^ Number of registered HN protein sequences assigned for comparison.

^*d*^ GenBank accession nos. QZB59302 (D) and BBL33137 (N).

^*e*^ Bold entries show amino acid mutations with no variation that were unique to the five passages.

^*f*^ NA, not analyzed due to insufficient coverage; -, not detected.

**TABLE 3:** List of nucleotide and amino acid mutations other than HN protein with >50% variant frequency

Gene	Culture (passage)	Name	Nucleotide	Reference	Allele	Frequency			Amino acid	Reference	Mutation
						P1	P3	P5			
P	HBTEC-ALI	PIV3_Fukushima_O726_2019 (C protein)	2090	G	C	-*^a^*	17.2	52.9	103 (99)	E (R)	Q (S)
	Vero/TMPRSS2	PIV1_Fukushima_O425_2018	1977	A	C	-	30.1	66.6	53	I	L
		PIV3_Fukushima_O644_2019	2420	G	A	-	86.7	70.8	210	D	N
M	Vero/TMPRSS2	PIV1_Fukushima_O425_2018	4273	G	A	-	70.4	68.6	210	V	M
		PIV2_Fukushima_O861_2019	3832	G	A	-	60.7	52.9	119	S	N
F	Vero/TMPRSS2	PIV3_Fukushima_O644_2019	5242	A	G	-	11.6	68.4	57	I	M
L	Vero/TMPRSS2	PIV1_Fukushima_H171_2018	14571	A	G	-	80.2	99.4	1941	R	G
		PIV1_Fukushima_O425_2018	13979	A	G	-	22.9	64.5	1744	E	G
		PIV1_Fukushima_O503_2019	14055	A	C	-	58.0	82.8	1769	Q	H
		PIV1_Fukushima_O503_2019	14454	G	A	-	99.8	99.6	1902	M	I
		PIV2_Fukushima_O33_2018	14477	C	T	-	40.3	63.2	1894	S	L
		PIV2_Fukushima_O836_2019	12281	A	T	-	29.5	88.0	1165	E	V
	Vero	PIV2_Fukushima_O33_2018	13475	C	T	-	46.5	74.3	1560	P	L
		PIV2_Fukushima_O836_2019	11898	G	T	-	-	82.3	1037	K	N
		PIV2_Fukushima_O836_2019	15167	G	A	-	-	83.7	2127	G	D

^*a*^ -, not detected.

## DISCUSSION

We hypothesized a lower incidence of mutations in HBTEC-ALI compared with conventional culture, and a higher incidence of mutations in Vero compared with Vero/TMPRSS2 cells, involving protease cleavage in HPIV replication, similar to coronaviruses (14). However, the mutation rate in Vero cells was not higher than in Vero/TMPRSS2 cells, suggesting that TMPRSS2 contributed little to HPIV2 infection of the cells. The F protein of trypsin-independent strains is thought to be cleaved by the ubiquitous cellular protease furin and can propagate in Vero cells (18). In the present study, the furin cleavage site of HPIV2 was identical to that of the trypsin-dependent strain, except that the amino acid at the P3 position was arginine (R) instead of glutamine (Q) (Table S6). This resulted in the furin-like cleavage motif RRER being similar to the spike polybasic site RRAR of SARS-CoV-2 (20). Although different from the furin cleavage motif R-X-R/K-R, the polybasic motif may alter the cleavage efficiency of F protein in Vero cells and contribute to the proliferation of HPIV2. In contrast, the mutation rate of the *L* gene was higher than that of HBTEC-ALI in the conventional cultured cells (Fig. 1H). The L protein of paramyxoviruses interacts with host chaperone proteins, and it is said to be important for the folding and stabilization of the L protein (21, 22). Adaptation to host proteins interacting with L protein could be related to mutations of *L* in the conventional cultured cells. Further studies are required to generate conventional cultured cells with low mutagenesis for HPIVs.

As well as in Vero/TMPRSS2 cells, the *HN* gene in HBTEC-ALI also showed a higher mutation rate than the other genes (Fig. 1D), which may be because the *HN* gene is more tolerant of nonsynonymous mutations (23). Although the nucleotide substitution rates of *HN* were different between HPIVs (24), it has been reported that *HN* gene has the next highest diversity after the *P* gene in HPIV1 (23). In addition, HN protein of clinical isolates of HPIV1 and HPIV2 showed extensive antigenic diversity (25, 26), which indicated that the flexibility could give HN the ability to escape the antibody response. Although mutations were seen during passage in HBTEC-ALI culture, they were at lower frequency than in conventional cultured cells. Such mutations could be avoided by using viruses with low passage history. Thus, HBTEC-ALI culture could be more useful for the study of all four HPIVs, as previously reported for HPIV3.

The HN protein has several important functions for viral entry and spread, such as receptor binding and activation of F protein and neuraminidase activity (27–29). The ectodomain of HN protein is composed of a helical stalk and a large globular head containing the sialic acid receptor binding and neuraminidase activity sites (28). Accumulation of the major amino acid mutations in HN globular domain in conventional cultured cells (Fig. 3) was similar to previous studies of the passage of HPIV3 clinical isolates (5). In those monolayer cultured cells with adapted HPIV3, increased receptor avidity and fusion promotion were reported (5–7). In the present study, more fusogenic isolates tended to be selected at P5 in HPIV1 and HPIV4 as well as HPIV3 (Table 1), suggesting that functional changes in HN could be a common problem in HPIVs. In HPIV3, HN variants with high fusogenicity have been reported to be disadvantaged for growth in human airway epithelial and *in vivo* fitness (30). Further analysis of the effect of the laboratory passage-associated mutation of HPIV1, 2, and 4 on HN function is necessary.

The HN dimer interface mutation N556D of HPIV3 in Vero/TMPRSS2 cells (Table 2; Fig. 3) was identical to that reported in other studies (5). The dimer interface mutations were enhanced and associated with higher fusogenicity in cultured cells (5, 31). Passage of PIV3_Fukushima_O330_2018 in Vero/TMPRSS2 cells caused N556D mutation, but syncytial formation was not observed at P5 (Table 1). Neither of the amino acid residues that weaken fusion activity, Q559R (6, 9) and R212L (32), were found in this isolate; therefore, other amino acids residues may be involved in counteracting the enhanced fusion activity of N556D. The identical laboratory passage-derived D216N mutation (Table 2; Fig. 3) has also been reported previously (31). These identical findings from completely independent studies strongly suggest that the mutation in HPIV3 caused by laboratory passage in cultured cells depends on selective pressure of conventional culture.

Quasispecies are thought to be a characteristic of clinical specimens, such as oropharyngeal/nasopharyngeal swabs of respiratory viruses, which play a significant role in disease development and clearance of respiratory viruses (32–34). Quasispecies variations in clinical specimens isolated from patients with human coronavirus OC43 have been identified in the S1 subunit of the spike protein that interacts with the receptor (35). Furthermore, patients with persistent SARS-CoV-2 infection are reported to show more quasispecies than patients with nonpersistent infection (34). In HPIV3, the characteristics of the variants of persistent infection in immunocompromised patients are reported to be similar to those of passage-related variants in cultured cells (32). These results indicate that, when using conventional cultured cells, it cannot be distinguished whether the variant is generated by passage or correctly represents the virus *in vivo*. D216N and N556D, which seemed to be passage-associated mutations, were also registered as sequences of clinical samples (Table 2). In addition, 192G and 556N (Table 2) have been reported as positively selected sites in molecular evolutionary studies of HPIV3 using conventional cultured cells (36). These mutations should be interpreted with caution in the context of molecular epidemiology, like the D151G/N mutation in IAV neuraminidase (12).

In conclusion, the present study demonstrated a high risk of *HN* mutagenesis during passage of HPIV1–HPIV4 clinical isolates in conventional cultured cells as previously reported for HPIV3. Mutation of *HN* could result in a high variant frequency even after a single passage. In addition, the L protein was more frequently mutated in HPIV1 and HPIV2 in conventional cultured cells than in HBTEC-ALI culture. Although Vero/TMPRSS2 cells were able to propagate HPIVs well, they are considered inappropriate for passage of clinical isolates. HBTEC-ALI culture appears to be more suitable than conventional culture for maintaining the original sequence and characteristics of clinical isolates of all four HPIVs.

## MATERIALS AND METHODS

### Cells

Vero cells (ATCC CCL81) were maintained in Dulbecco’s modified Eagle’s medium-high glucose (DMEM; D5796, Sigma, St. Louis, MO, USA) containing 5% fetal calf serum (FCS; F7524, Sigma) and penicillin-streptomycin (168-23191, FUJIFILM Wako Pure Chemical Corporation, Osaka, Japan). Vero/TMPRSS2 cells (37) were maintained in DMEM containing 10% FCS, 1 mg/mL G418 sulfate (ALX-380-013-G005, Enzo Life Sciences, Farmingdale, NY, USA) and penicillin-streptomycin. HBTE cells (FC-0035, Lifeline Cell Technology, Frederick, MD, USA) were purchased and the same lots (#01335) were used throughout the study. HBTEC-ALI culture was prepared as described previously (38). Expansion of HBTE cells in submerged culture was performed with a 1:1 mixture of PneumaCult-Ex Medium (05008, STEMCELL Technologies, Vancouver, Canada) and PneumaCult-Ex Plus Medium (05040, STEMCELL Technologies) supplemented with hydrocortisone (07904, STEMCELL Technologies). Expanded HBTE cells were removed using EDTA-trypsin solution (0.02% EDTA, 0.1% trypsin) (Cosmo Bio, Tokyo, Japan) and trypsin inhibitor (T6522, Sigma). A total of 10^5^ cells per insert were seeded into the collagen-coated (Cellmatrix Type IV; Nitta Gelatin, Osaka, Japan) apical chamber of a Costar 6.5 mm Transwell with 0.4 µm pore polyester membrane insert (3470, Corning, Kennebunk, ME, USA). After a few days, culture HBTE cells were generated by ALI culture using PneumaCult-ALI Medium (05001, STEMCELL Technologies) supplemented with heparin solution (07980, STEMCELL Technologies) and hydrocortisone. After at least 3 weeks, well-differentiated cells were used in the experiments. Cells were cultured in a 5% CO_2_ incubator at 37°C until virus inoculation.

### Viruses

All P0 isolates of HPIVs were obtained using HBTEC-ALI culture from clinical specimens from pediatric inpatients collected in previous studies (39). HPIV-qPCR-positive nasopharyngeal swabs were diluted two- to fourfold with DMEM containing 1% FCS, inoculated into the well-differentiated HBTEC-ALI cultures at a volume of 100 µL, and incubated at 34°C in 5% CO_2_. One specimen was inoculated into one HBTEC-ALI culture. After overnight incubation, the apical surface was washed four times and ALI culture was continued. At 4, 7, and 11 days post-inoculation (dpi), the apical surface was washed and the medium in the bottom well was refreshed. A total of 400 µL washing solution (100 µL × four times with DMEM containing 1% FCS) was obtained from each day and stored at −80°C. The apical washing solutions from 7 or 11 dpi were used as P0 isolates. The sequences of the P0 isolates were as follows: HPIV1 (GenBank accession nos. LC764862, LC764863, LC764864, and LC764865), HPIV2 (LC720864, LC720865, LC720868, and LC720869), HPIV3 (LC720876, LC720877, and LC720879), and HPIV4 (LC706553, LC706554, and LC706556) (40, 41).

### Blind passages of HPIVs

For Vero and Vero/TMPRSS2 cell passages, cells plated the previous day in 24-well multiplates were used. Cells were inoculated with fivefold-diluted P0 isolates and incubated in a 5% CO_2_ incubator at 34°C. After 24-h incubation, cells were washed three times, and fresh medium was added (500 µL DMEM containing 2% FCS). At 3–8 dpi, the culture medium (P1 solution) was harvested and inoculated into newly prepared cells for P2 passage. Inoculation was performed at a 100-fold dilution if a CPE was obvious, and at a twofold dilution if not. These procedures were continued until P5 solutions were obtained. For HBTEC-ALI passages, well-differentiated cells were used. A fivefold dilution of P0 isolates was inoculated into the insert in a volume of 100 µL and incubated in a 5% CO_2_ at 34°C. After 24-h incubation, the apical surface was washed four times and ALI culture was continued. At 4 dpi, the medium in the bottom well was refreshed. At 7 dpi, 400 µL apical washing solution (P1, 200 µL × two times with DMEM containing 1% FCS) was harvested and inoculated into previously prepared, new HBTEC-ALI for P2 passage. Inoculation was performed at a fivefold dilution in a volume of 100 µL. These procedures were continued until P5 solutions were obtained. All viral solutions were centrifuged after harvesting at 2,300 *g* at 4°C for 15 min, and the supernatant was used for inoculation and stored at −80°C. Antimycoplasma drug KM881012 (KAC, Hyogo, Japan) was used as needed.

### RNA extraction

For NGS, before RNA extraction, Ribonuclease (DNase-free) Glycerol Solution (Nippon Gene, Tokyo, Japan) was used to remove RNA from outside the viral particles. P1, P3, and P5 solutions were subjected to RNA extraction using ISOGEN-LS reagent (311-02621, Nippon Gene). For identification of viral growth, nucleic acids were extracted using QIAamp 96 Virus QIAcube HT kit (Qiagen, Hilden, Germany) following the manufacturer’s instructions, except that the elusion step was performed by centrifugation.

### Real-time PCR for detection of HPIVs

Reverse transcription was performed using SMART M-MLV Reverse Transcriptase (Takara Bio, Shiga, Japan) with random primers (11034731001, Roche, Basel, Switzerland). Real-time PCR was performed using LightCycler 480 Probes Master (Roche) and LightCycler 96 or LightCycler 480 II instrument (Roche) under the following conditions: 95°C for 10 min and 45 cycles of 95°C for 10 s and 60°C for 30 s (acquisition). The final concentrations of primers and probe were 900 nM and 200 nM, respectively. The primer and probe sequences for HPIV1–HPIV3 were the same as previously reported (42), and those for HPIV4 were modified (43) as follows: HPIV1 forward, 5′-CCATCCTTTTTCTGCAATGTATCC-3′; HPIV1 reverse, 5′-ATTGCAAACACTCTGATTAACATTGG-3′; HPIV1 probe, 5′-Cy5- CGGTGGCTTAACAACTCCGCTCCAAGG-BHQ3-3′; HPIV2 forward, 5′-GGACGCCTAAATATGGACCTCTC-3′; HPIV2 reverse, 5′-GTGAGTGTAACACCAATGGGTCT-3′; HPIV2 probe, 5′-Cy5-CCCAGCTTTATCCCCTCAGCAACATCTCCC-BHQ3-3′; HPIV3 forward, 5′-ATGGACATGGCATAATGTGCTAT-3′; HPIV3 reverse, 5′-AATGCTYCCTGTGGGATTGAG-3′; HPIV3 probe, 5′-FAM-TCCCCATGGACATTCATTGTTTCCTGGTCT-BHQ1-3′; HPIV4 forward, 5′-CAAAYGATCCACAGCAAAGATTC-3′; HPIV4 reverse, 5′-ATGTGGCCTGTAAGGAAAGCA-3′; HPIV4 probe, 5′-VIC-GTATCATCATCTGCCAAAT-MGB-3′.

### NGS

NGS libraries were prepared using NEBNext Ultra II RNA Library Prep Kit for Illumina (E7770S, New England Biolabs, Ipswich, MA, USA) and NEBNext Multiplex Oligos for Illumina (96 Index Primers) (E6609S, NEB), and purified with AMPure XP magnetic beads (A63881, Beckman Coulter, Brea, CA, USA). The quality of the purified library was assessed on an MCE-202 MultiNA (Shimadzu Corporation, Kyoto, Japan) using DNA-1000 Reagent Kit (S292-27911-91, Shimadzu) and SYBR Gold Nucleic Acid Gel Stain (S11494, Invitrogen, Waltham, MA, USA). The concentration was determined on a Quantus Fluorometer (Promega Corporation, Madison, WI, USA) with QuantiFluor dsDNA System (E2670, Promega). The libraries were normalized and pooled until sequencing at−30°C. A 76-cycle paired-end read sequencing was carried out on a MiSeq (Illumina, San Diego, CA, USA) using the MiSeq Reagent Kit v3 (150-cycle) (Illumina) at our laboratory, or on a DNBSEQ-G400 instrument at GENEWIZ (South Plainfield, NJ, USA).

### Variant detection and mutation analysis

NGS data were manipulated using CLC Genomics Workbench v.22.0.2 or v.23.0.4 (QIAGEN). P0 isolate sequences were generated by *de novo* assembly and mapping to related sequences. Virus genomes were annotated using VAPiD v.1.6.6 or v.1.6.7 (44). Quasispecies that exist at low frequencies cannot be distinguished from sequencing errors (45). Therefore, a cutoff of 1% frequency was set for the quasispecies in the P0 inoculum to avoid missing low-frequency quasispecies and to minimize the impact of errors. Variants were detected for P0 isolates using a low-frequency variant detection tool in the setting of 1% minimum frequency, which were defined as quasispecies (Table S5). Variants were also detected for P1, P3, and P5 viruses against corresponding P0 sequences using the basic variant detection tool in the setting of 10% minimum frequency, which were defined as nucleotide substitutions (Tables S2 through S4). Mutations in P1, P3, and P5 viruses were defined as those in which quasispecies were excluded from the nucleotide substitutions. Mutation rate in P5 viruses was obtained by dividing the number of mutations by the genome length. Similarly, the mutation rate of a gene in a P5 virus was determined by dividing the number of mutations by the gene length.

### Analysis of the amino acid mutations in HN and L proteins

The structural positions of the amino acid residues of mutations were estimated by superimposing the sequence of HPIVs on the HN ectodomain structure of a closely related virus (PDB: 4JF7, parainfluenza virus 5) (28), or the L-P complex structure (PDB: 6V85, parainfluenza virus 5) (19), using the SWISS-MODEL server (46) and the PyMOL Molecular Graphics System, version 2.6.0a0 Open-Source (Schrödinger, LLC). To analyze the amino acid variations of the mutation sites, all available HN protein sequences of HPIVs were collected from the NCBI protein database (accessed on 2 June 2023). The amino acid sequences were aligned and analyzed using MAFFT online service (MAFFT version 7, https://mafft.cbrc.jp/alignment/server/) (47) and MEGA software (version 11.0.3) (48).

### Statistics

One-way analysis of variance and Tukey’s multiple comparison test were performed for comparison of the mutation rate using GraphPad Prism 8 software version 8.4.3 (GraphPad Software, La Jolla, CA, USA).

## Data Availability

The raw reads were deposited under BioProject number PRJDB18043.

## SUPPLEMENTAL MATERIAL

Supplemental figures and tables spectrum.01164-24-s0001.pdf Fig. S1 and S2; Tables S1-S6.

ASM does not own the copyrights to Supplemental Material that may be linked to, or accessed through, an article. The authors have granted ASM a non-exclusive, world-wide license to publish the Supplemental Material files. Please contact the corresponding author directly for reuse.
