# Supplementary material for: Comparison of mutations in human parainfluenza viruses during passage in primary human bronchial/tracheal epithelial air-liquid interface cultures and cell lines
Source: Microbiol Spectr. 2024 Jul 30;12(9):e01164-24. doi: 10.1128/spectrum.01164-24 (PMC11370246; doi:10.1128/spectrum.01164-24)
Supplement: Supplemental figures and tables — Fig. S1 and S2; Tables S1-S6. [file spectrum.01164-24-s0001.pdf]

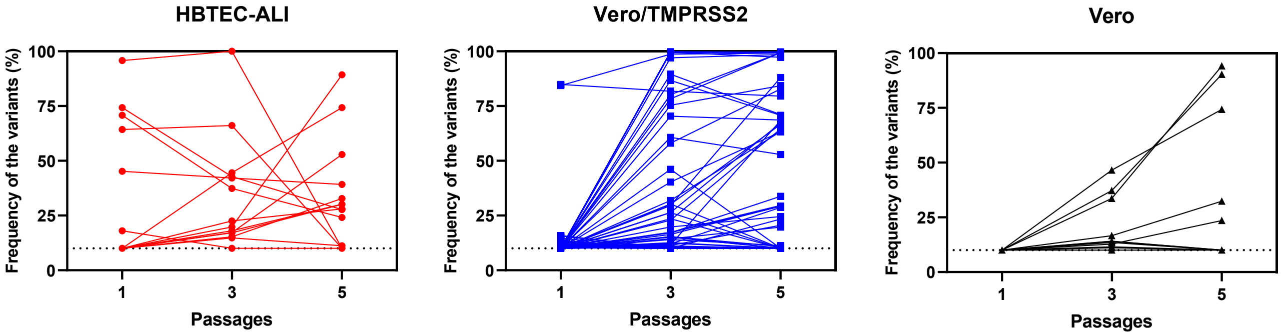

Fig. S1. Change in the variant frequency occurred at P1 and P3.

The nucleotide mutations which occurred at P1 and P3 were followed and the change in the variant frequency of each mutation were shown in a line. The dotted line represents 10% of the detection threshold.

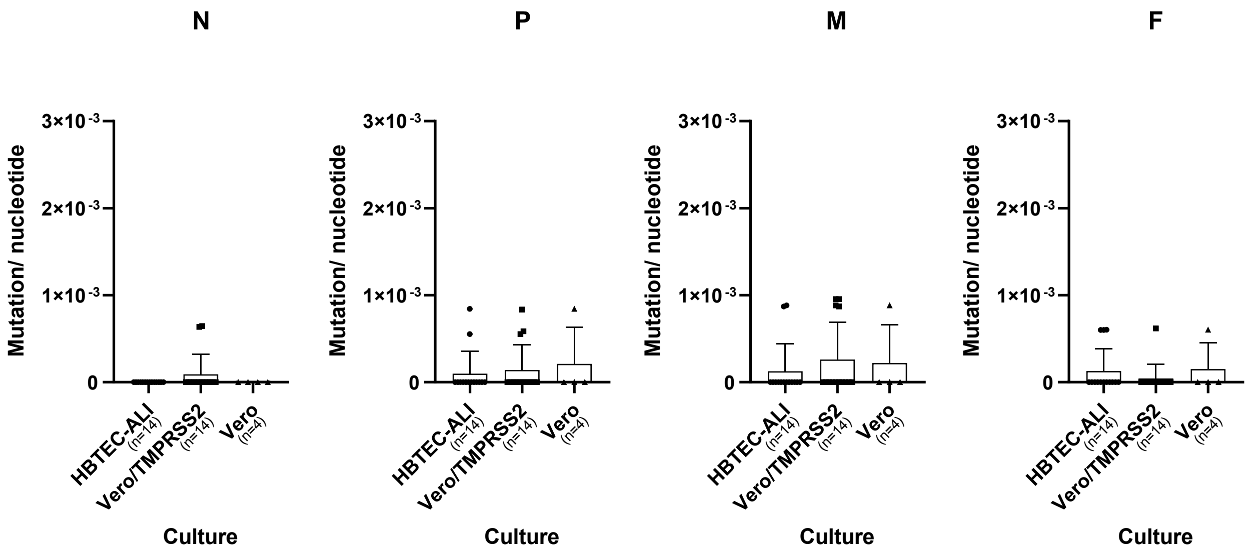

Fig. S2. Mutation rate in each gene.

The mutation rate of each gene after five blind passages in different cultures. "n" indicates the number of isolates used in the study. Statistics were calculated using Tukey's multiple comparisons test (*P<0.05), and there were no significant difference in *N, P, M* and *F*.

Table S1. Viral genome copy numbers at each passage

			Log (genome copy numbers/mL)									
Culture (passage)	Species	Name	Wash	P1	Wash	P2	Wash	P3	Wash	P4	Wash	P5
HBTEC-ALI	Respirovirus laryngotracheitidis	PIV1_Fukushima_O77_2018 (LC764863)	3.6	8.2	4.6	7.5	4.8	8.1	4.3	6.6	3.7	7.3
	(Human parainfluenza virus 1)	PIV1_Fukushima_H171_2018 (LC764862)	3.4	7.5	3.0	6.6	4.4	8.9	4.5	7.2	4.0	8.3
		PIV1_Fukushima_O425_2018 (LC764864)	3.4	7.5	3.4	6.2	4.4	8.8	4.3	7.2	3.9	8.5
		PIV1_Fukushima_O503_2019 (LC764865)	4.1	8.3	4.4	6.7	4.8	8.6	4.9	7.3	4.5	9.1
	Orthorubulavirus laryngotracheitidis	PIV2_Fukushima_O13_2018 (LC720864)	ND	10.1	2.9	8.7	6.3	9.6	6.2	8.3	5.9	9.3
	(Human parainfluenza virus 2)	PIV2_Fukushima_O33_2018 (LC720865)	ND	10.0	6.3	9.1	6.2	9.6	5.7	8.5	4.5	9.3
		PIV2_Fukushima_O836_2019 (LC720868)	5.2	9.9	6.0	9.0	6.1	9.5	5.8	8.4	4.5	9.3
		PIV2_Fukushima_O861_2019 (LC720869)	5.3	9.9	5.5	9.1	5.9	9.9	5.7	9.4	5.6	10.1
	Respirovirus pneumoniae	PIV3_Fukushima_O330_2018 (LC720876)	5.8	11.1	7.9	9.5	7.5	11.0	7.5	9.2	5.8	10.7
	(Human parainfluenza virus 3)	PIV3_Fukushima_O644_2019 (LC720877)	5.1	10.9	7.2	9.7	7.4	11.0	7.4	9.4	6.0	10.8
		PIV3_Fukushima_O726_2019 (LC720879)	ND	10.5	6.8	9.4	7.0	10.7	7.1	9.3	5.9	10.5
	Orthorubulavirus hominis	PIV4a_Fukushima_H725_2019 (LC706553)	3.3	6.4	ND	6.6	3.2	8.6	4.4	7.1	2.6	8.2
	(Human parainfluenza virus 4)	PIV4b_Fukushima_O896_2019 (LC706554)	2.6	7.8	3.7	7.2	3.8	7.9	3.1	6.2	2.6	7.8
		PIV4b_Fukushima_OR476_2022 (LC706556)	3.4	8.1	3.4	7.9	2.9	8.6	3.5	7.6	ND	9.0
Vero/TMPRSS2	Respirovirus laryngotracheitidis	PIV1_Fukushima_O77_2018 (LC764863)	5.0	6.3	4.4	5.8	4.8	6.1	4.7	5.4	5.0	6.0
	(Human parainfluenza virus 1)	PIV1_Fukushima_H171_2018 (LC764862)	4.2	6.4	NT	6.2	NT	7.6	NT	7.5	6.0	8.1
		PIV1_Fukushima_O425_2018 (LC764864)	4.1	6.3	NT	6.8	NT	7.6	NT	7.5	5.5	8.0
		PIV1_Fukushima_O503_2019 (LC764865)	3.8	6.4	NT	6.9	NT	8.8	NT	6.2	4.3	7.7
	Orthorubulavirus laryngotracheitidis	PIV2_Fukushima_O13_2018 (LC720864)	6.5	8.7	4.3	9.0	6.0	9.0	4.6	8.2	5.6	8.4
	(Human parainfluenza virus 2)	PIV2_Fukushima_O33_2018 (LC720865)	6.2	8.6	3.8	8.9	5.5	9.1	3.9	9.0	5.6	8.6
		PIV2_Fukushima_O836_2019 (LC720868)	6.5	8.6	ND	8.6	5.7	9.0	4.9	7.9	5.7	8.5
		PIV2_Fukushima_O861_2019 (LC720869)	6.0	7.9	NT	8.3	NT	8.6	NT	8.5	6.2	8.9
	Respirovirus pneumoniae	PIV3_Fukushima_O330_2018 (LC720876)	7.2	8.3	7.5	10.2	7.9	11.0	7.8	10.6	7.8	10.8
	(Human parainfluenza virus 3)	PIV3_Fukushima_O644_2019 (LC720877)	6.1	7.7	6.8	8.8	5.7	9.0	5.5	8.4	6.5	9.6
		PIV3_Fukushima_O726_2019 (LC720879)	7.1	9.6	5.3	10.3	7.9	10.7	7.3	10.4	8.0	10.7
	Orthorubulavirus hominis	PIV4a_Fukushima_H725_2019 (LC706553)	4.4	5.1	NT	4.8	NT	7.1	NT	6.0	3.8	7.0
	(Human parainfluenza virus 4)	PIV4b_Fukushima_O896_2019 (LC706554)	3.3	4.1	ND	4.7	4.6	6.6	3.2	6.4	3.1	7.5
		PIV4b_Fukushima_OR476_2022 (LC706556)	5.1	7.6	NT	6.4	NT	7.6	NT	5.9	4.0	7.2
Vero	Orthorubulavirus laryngotracheitidis	PIV2_Fukushima_O13_2018 (LC720864)	5.4	7.1	6.1	7.4	6.3	8.2	ND	6.6	5.9	8.1
	(Human parainfluenza virus 2)	PIV2_Fukushima_O33_2018 (LC720865)	5.8	7.8	NT	7.7	NT	8.1	NT	9.1	5.9	9.2
		PIV2_Fukushima_O836_2019 (LC720868)	5.2	7.1	6.0	7.1	6.6	8.2	ND	5.7	4.7	7.9
		PIV2_Fukushima_O861_2019 (LC720869)	6.2	8.2	NT	8.4	NT	8.7	NT	8.8	5.8	9.1

ND: Not detected, NT: Not tested.

Table S2. List of the nucleotide substitutions that found in the P1 solution at >10% frequency, including location, coverage and frequency

Species	Name (Genbank ID)	Culture (passage)	Region	Gene	Type	Reference	Allele	Coverage	Frequency
Respirovirus laryngotracheitidis	PIV1_Fukushima_O77_2018 (LC764863)	HBTEC-ALI	**8840**	L	Silent	T	C	37658	19.6
(Human parainfluenzavirus 1)		Vero/TMPRSS2	**8840**	L	Silent	T	C	2455	19.7
	PIV1_Fukushima_H171_2018 (LC764862)	HBTEC-ALI	**3012**	P	Missense	A	G	48131	25.7
		Vero/TMPRSS2	**3012**	P	Missense	A	G	924	27.7
	PIV1_Fukushima_O425_2018 (LC764864)	HBTEC-ALI	Not detected						
		Vero/TMPRSS2	**4**	-	SNV	G	A	86	10.5
			**7377**	HN	Silent	C	T	1107	10.0
			**8448**	HN	Missense	T	A	1974	17.1
	PIV1_Fukushima_O503_2019 (LC764865)	HBTEC-ALI	**5492**	F	Missense	C	T	65235	84.4
		Vero/TMPRSS2	**5492**	F	Missense	C	T	1667	46.1
Orthorubulavirus laryngotracheitidis	PIV2_Fukushima_O13_2018 (LC720864)	HBTEC-ALI	Not detected						
(Human parainfluenzavirus 2)		Vero/TMPRSS2	Not detected						
		Vero	Not detected						
	PIV2_Fukushima_O33_2018 (LC720865)	HBTEC-ALI	Not detected						
		Vero/TMPRSS2	Not detected						
		Vero	Not detected						
	PIV2_Fukushima_O836_2019 (LC720868)	HBTEC-ALI	**13665**	L	Silent	T	C	97316	20.0
		Vero/TMPRSS2	**13665**	L	Silent	T	C	46258	20.4
		Vero	**13665**	L	Silent	T	C	4462	20.9
	PIV2_Fukushima_O861_2019 (LC720869)	HBTEC-ALI	Not detected						
		Vero/TMPRSS2	Not detected						
		Vero	Not detected						
Respirovirus pneumoniae	PIV3_Fukushima_O330_2018 (LC720876)	HBTEC-ALI	**6184**	F	Silent	T	C	46905	24.7
(Human parainfluenzavirus 3)		Vero/TMPRSS2	**6184**	F	Silent	T	C	235	20.9
			10610	L	Silent	T	C	128	10.2
	PIV3_Fukushima_O644_2019 (LC720877)	HBTEC-ALI	10	-	SNV	A	G	24	95.8
		Vero/TMPRSS2	1358	N	Silent	C	A	220	10.9
			1463	N	Silent	G	A	173	11.6
			2109	P	Missense	C	T	69	13.0
			2147	P	Missense	A	G	74	12.2
			2539	P	Missense	G	A	141	15.6
			3072	P	Missense	C	T	66	10.6
			**3427^3428**	P	Insertion	-	A	37	10.8
			4055	M	Silent	A	G	87	11.5
			4316	M	Silent	G	A	84	10.7
			4769	M	Silent	C	T	235	13.6
			4819	-	SNV	T	C	223	10.8
			4926	-	SNV	T	G	58	12.1
			5029	-	SNV	G	A	62	11.3
			5108	F	Missense	G	A	41	14.6
			5936	F	Silent	T	C	82	15.9
			6885	HN	Missense	G	A	145	10.3
			6910	HN	Silent	C	A	149	10.7
			7588	HN	Silent	G	A	112	10.7
			8687	L	Silent	T	C	92	12.0
			9839	L	Silent	C	T	60	11.7
			9860	L	Silent	T	C	54	13.0
			9971	L	Silent	A	G	39	10.3
			10436	L	Silent	A	G	47	10.6
			10964	L	Silent	T	C	82	12.2
			11858	L	Silent	C	T	83	10.8
			11873	L	Silent	C	T	87	12.6
			14763	L	Missense	A	G	90	11.1
			15441^15442	-	Insertion	-	A	33	12.1
	PIV3_Fukushima_O726_2019 (LC720879)	HBTEC-ALI	**6900**	HN	Missense	T	C	44080	11.2
			**6964**	HN	Silent	T	C	81868	10.1
		Vero/TMPRSS2	**6900**	HN	Missense	T	C	2380	88.5
			**6964**	HN	Silent	T	C	3494	87.8
			7451	HN	Missense	G	A	2481	84.4
Orthorubulavirus hominis	PIV4a_Fukushima_H725_2019 (LC706553)	HBTEC-ALI	4	-	SNV	T	A	210	64.3
(Human parainfluenzavirus 4)			**5491**	F	Silent	T	C	16972	13.8
			**5497**	F	Silent	T	C	16487	13.4
			**5821**	F	Silent	C	T	31635	21.2
			**9237**	HN	Missense	A	G	29676	63.0
			**16235^16236**	L	Insertion	-	T	9072	10.9
		Vero/TMPRSS2	* Not analyzed						
	PIV4b_Fukushima_O896_2019 (LC706554)	HBTEC-ALI	3736	M	Silent	G	A	56590	74.3
			**6450**	F	Silent	T	C	38750	99.7
			**7536**	-	SNV	A	G	29994	99.6
			9232	HN	Missense	G	A	35536	70.8
			17353^17354	-	Insertion	-	CTTGGT	1215	45.2
			17353^17354	-	Insertion	-	CTTGGTAT	1215	18.0
		Vero/TMPRSS2	* Not analyzed						
	PIV4b_Fukushima_OR476_2022 (LC706556)	HBTEC-ALI	**8561**	HN	Silent	A	G	42320	37.9
			**9119**	HN	Silent	C	T	54171	36.8
		Vero/TMPRSS2	**2419**	P	Missense	A	T	2500	98.3
			**8152**	HN	Missense	G	A	3019	98.6

9022	HN	Missense	C	A	4449	84.9

N; nucleocapsid protein, P; phosphoprotein, M; matrix protein, F; fusion glycoprotein, HN; hemagglutinin-neuraminidase glycoprotein, L; large protein.

Bold; quasispecies present in both P0 and P1.

* Unable to analyze due to insufficient average coverage of <100x.

Table S3. List of the nucleotide substitutions that found in the P3 solution at >10% frequency, including location, coverage and frequency

Species	Name (Genbank ID)	Culture (passage)	Region	Gene	Type	Reference	Allele	Coverage	Frequency
Respirovirus laryngotracheitidis	PIV1_Fukushima_O77_2018 (LC764863)	HBTEC-ALI	6	-	SNV	T	A	1535	15.3
(Human parainfluenzavirus 1)			**8840**	L	Silent	T	C	52235	23.3
		Vero/TMPRSS2	8453	HN	Missense	T	A	2836	15.3
			**8840**	L	Silent	T	C	1583	19.1
	PIV1_Fukushima_H171_2018 (LC764862)	HBTEC-ALI	**3012**	P	Missense	A	G	35674	23.6
			**8277**	HN	Missense	T	C	89119	21.0
		Vero/TMPRSS2	515	N	Silent	C	T	6015	12.4
			**3012**	P	Missense	A	G	4581	95.4
			7531	HN	Missense	C	T	2530	78.3
			14571	L	Missense	A	G	4315	80.2
	PIV1_Fukushima_O425_2018 (LC764864)	HBTEC-ALI	**4**	-	SNV	G	A	1081	31.6
			**7377**	HN	Silent	C	T	33062	52.3
		Vero/TMPRSS2	939	N	Missense	G	A	17660	12.0
			1977	P	Missense	A	C	15753	30.1
			4273	M	Missense	G	A	11003	70.4
			7291	HN	Missense	T	C	10496	16.4
			8193	HN	Silent	A	T	10802	31.9
			8236	HN	Missense	A	G	11314	26.4
			**8448**	HN	Missense	T	A	9743	70.2
			13979	L	Missense	A	G	5336	22.9
	PIV1_Fukushima_O503_2019 (LC764865)	HBTEC-ALI	**5492**	F	Missense	C	T	71828	86.9
			8449	HN	Missense	G	A	87206	44.5
		Vero/TMPRSS2	7646	HN	Missense	A	G	20711	99.6
			12450	L	Silent	C	T	70974	99.8
			14055	L	Missense	A	C	76960	58.0
			14454	L	Missense	G	A	80190	99.8
Orthorubulavirus laryngotracheitidis	PIV2_Fukushima_O13_2018 (LC720864)	HBTEC-ALI	Not detected						
(Human parainfluenzavirus 2)		Vero/TMPRSS2	9239	L	Missense	G	A	22574	10.7
			14477	L	Missense	C	T	18091	10.5
			14684	L	Missense	G	A	15067	12.6
		Vero	2638	P	Missense	C	T	18749	11.5
			7663	HN	Missense	C	T	48794	14.0
			14477	L	Missense	C	T	16224	16.6
	PIV2_Fukushima_O33_2018 (LC720865)	HBTEC-ALI	14	-	SNV	T	C	17898	19.8
		Vero/TMPRSS2	12344	L	Missense	G	A	7955	15.4
			14477	L	Missense	C	T	9199	40.3
		Vero	13475	L	Missense	C	T	21853	46.5
			14093	L	Missense	C	T	18824	12.8
			14684	L	Missense	G	A	12439	10.0
	PIV2_Fukushima_O836_2019 (LC720868)	HBTEC-ALI	Not detected						
		Vero/TMPRSS2	12281	L	Missense	A	T	15943	29.5
			12335	L	Missense	G	A	17986	15.1
		Vero	2513	P	Missense	G	A	19178	14.1
			12335	L	Missense	G	A	10400	11.1
			**13665**	L	Silent	T	C	31723	17.8
	PIV2_Fukushima_O861_2019 (LC720869)	HBTEC-ALI	4593	M	Silent	T	C	45589	17.1
			5163	F	Missense	C	T	70435	18.0
		Vero/TMPRSS2	3832	M	Missense	G	A	15355	60.7
			14518	L	Missense	G	T	13340	19.8
			15292	L	Silent	G	A	19092	11.4
		Vero	7404	HN	Missense	A	G	23403	33.6
			13615	L	Silent	T	C	43389	37.2
			14933	L	Missense	G	A	31065	13.5
Respirovirus pneumoniae	PIV3_Fukushima_O330_2018 (LC720876)	HBTEC-ALI	**6184**	F	Silent	T	C	57749	26.1
(Human parainfluenzavirus 3)		Vero/TMPRSS2	**6184**	F	Silent	T	C	9691	99.6
			8471	HN	Missense	A	G	3132	99.4
	PIV3_Fukushima_O644_2019 (LC720877)	HBTEC-ALI	10	-	SNV	A	G	26	100.0
			**28**	-	SNV	A	T	2851	10.5
		Vero/TMPRSS2	2420	P	Missense	G	A	708	86.7
			5242	F	Missense	A	G	473	11.6
			7493	HN	Missense	A	G	1048	89.5
	PIV3_Fukushima_O726_2019 (LC720879)	HBTEC-ALI	2090	P	Missense	G	C	23768	17.2
			**6900**	HN	Missense	T	C	36053	14.4
			**6964**	HN	Silent	T	C	61608	13.3
			12916	L	Missense	T	C	23211	14.8
		Vero/TMPRSS2	**6900**	HN	Missense	T	C	4594	99.1
			**6964**	HN	Silent	T	C	6107	99.2
			7379	HN	Missense	A	G	4303	97.0
			7451	HN	Missense	G	A	4094	98.7
Orthorubulavirus hominis	PIV4a_Fukushima_H725_2019 (LC706553)	HBTEC-ALI	4	-	SNV	T	A	481	66.1
(Human parainfluenzavirus 4)			**9237**	HN	Missense	A	G	49843	15.2
			**16235^16236**	L	Insertion	-	T	18046	12.4
		Vero/TMPRSS2	**4110**	M	Missense	A	G	5731	98.8
			**5821**	F	Silent	C	T	2900	99.8
			8049	HN	Missense	A	T	3007	75.4
			**9237**	HN	Missense	A	G	3374	99.3
	PIV4b_Fukushima_O896_2019 (LC706554)	HBTEC-ALI	3736	M	Silent	G	A	62724	42.8

		**6450**	F	Silent	T	C	45282	99.6
		**7536**	**-**	SNV	A	G	33857	99.6
		9232	HN	Missense	G	A	41807	37.4
		17353^17354	**-**	Insertion	-	CTTGGT	2455	42.1
	Vero/TMPRSS2	**758**	N	Silent	G	A	2887	10.4
		2637	P	Missense	T	C	3042	11.1
		**3454**	-	SNV	A	G	3862	10.1
		3736	M	Silent	G	A	4171	30.3
		**4276**	M	Silent	C	T	4870	10.3
		**6450**	F	Silent	T	C	2785	90.3
		**7536**	**-**	SNV	A	G	2626	88.6
		9085	HN	Missense	A	G	4721	46.1
		9232	HN	Missense	G	A	2577	26.4
		17353^17354	**-**	Insertion	-	CTTGGT	81	23.5
PIV4b_Fukushima_OR476_2022 (LC706556)	HBTEC-ALI	**8561**	HN	Silent	A	G	26679	52.8
		9028	HN	Missense	C	T	59792	22.5
		**9119**	HN	Silent	C	T	58359	51.5
	Vero/TMPRSS2	**2419**	P	Missense	A	T	3156	99.2
		**8152**	HN	Missense	G	A	3192	99.1
		9022	HN	Missense	C	A	3978	81.8
		10479	L	Missense	G	T	1733	14.0
		16074	L	Missense	G	A	2434	14.4
		17094	-	SNV	A	G	13291	17.2
		17099	-	SNV	A	G	13389	17.4
		17166	-	SNV	A	G	12689	17.1

N; nucleocapsid protein, P; phosphoprotein, M; matrix protein, F; fusion glycoprotein, HN; hemagglutinin-neuraminidase glycoprotein, L; large protein.

Bold; quasispecies present in both P0 and P3.

Table S4. List of the nucleotide substitutions that found in the P5 solution at >10% frequency, including location, coverage and frequency

Species	Name (Genbank ID)	Culture (passage)	Region	Gene	Type	Reference	Allele	Coverage	Frequency
Respirovirus laryngotracheitidis	PIV1_Fukushima_O77_2018 (LC764863)	HBTEC-ALI	6	-	SNV	T	A	174	32.8
(Human parainfluenzavirus 1)			**8840**	L	Silent	T	C	4503	29.0
		Vero/TMPRSS2	4104	M	Missense	G	C	1447	32.5
			7394	HN	Missense	C	A	1094	27.1
			8542	HN	Missense	C	T	634	32.3
			**8840**	L	Silent	T	C	710	43.2
	PIV1_Fukushima_H171_2018 (LC764862)	HBTEC-ALI	**3012**	P	Missense	A	G	6620	35.9
			7304	HN	Silent	C	T	8192	13.2
			**8277**	HN	Missense	T	C	10996	18.2
			9592	L	Missense	G	A	7157	10.1
		Vero/TMPRSS2	**3012**	P	Missense	A	G	2960	99.6
			7531	HN	Missense	C	T	2094	99.4
			8421	HN	Missense	A	G	4508	94.6
			14571	L	Missense	A	G	3076	99.4
	PIV1_Fukushima_O425_2018 (LC764864)	HBTEC-ALI	**4**	-	SNV	G	A	101	70.3
			5740^5741	F	Insertion	-	A	6475	11.8
			**7377**	HN	Silent	C	T	3680	91.3
			11352	L	Silent	C	T	7564	10.4
		Vero/TMPRSS2	939	N	Missense	G	A	7350	28.7
			1977	P	Missense	A	C	8947	66.6
			4273	M	Missense	G	A	5042	68.6
			7291	HN	Missense	T	C	4402	29.3
			7292	HN	Missense	G	A	4417	34.2
			8193	HN	Silent	A	T	4435	63.4
			8236	HN	Missense	A	G	5125	67.3
			8281	HN	Missense	A	G	5060	28.9
			**8448**	HN	Missense	T	A	4225	68.5
			13979	L	Missense	A	G	2662	64.5
	PIV1_Fukushima_O503_2019 (LC764865)	HBTEC-ALI	**5492**	F	Missense	C	T	11498	92.0
			5740^5741	F	Insertion	-	A	7107	12.0
			8449	HN	Missense	G	A	10909	74.3
		Vero/TMPRSS2	7646	HN	Missense	A	G	1609	97.3
			12450	L	Silent	C	T	11820	99.3
			14055	L	Missense	A	C	12651	82.8
			14454	L	Missense	G	A	13267	99.6
Orthorubulavirus laryngotracheitidis	PIV2_Fukushima_O13_2018 (LC720864)	HBTEC-ALI	14	-	SNV	T	C	3773	49.9
(Human parainfluenzavirus 2)			14	-	SNV	T	G	3773	18.8
			7592	HN	Missense	C	T	30933	32.2
		Vero/TMPRSS2	7508	HN	Missense	A	G	4384	28.0
			8129	HN	Missense	C	T	10576	22.4
			10330	L	Missense	T	C	1681	28.6
			12344	L	Missense	G	A	2304	27.6
		Vero	11858	L	Missense	G	T	26939	12.0
			12072	L	Missense	G	A	20832	21.4
			14477	L	Missense	C	T	16193	32.4
	PIV2_Fukushima_O33_2018 (LC720865)	HBTEC-ALI	14	-	SNV	T	C	4495	89.3
		Vero/TMPRSS2	7112	HN	Missense	T	C	2383	17.2
			12344	L	Missense	G	A	797	10.9
			14477	L	Missense	C	T	1175	63.2
		Vero	5531	F	Missense	A	G	8359	18.7
			7535	HN	Missense	G	A	7832	72.1
			13475	L	Missense	C	T	6912	74.3
			14093	L	Missense	C	T	5183	23.5
	PIV2_Fukushima_O836_2019 (LC720868)	HBTEC-ALI	5	-	SNV	T	C	4576	36.5
			5	-	SNV	T	G	4576	39.1
			2164	P	Missense	C	T	49856	39.8
			**8441**	HN	Missense	T	A	7809	19.4
		Vero/TMPRSS2	7274	HN	Missense	G	A	2218	71.2
			12281	L	Missense	A	T	1811	88.0
		Vero	3857	M	Missense	G	A	33296	17.8
			7337	HN	Missense	A	G	15037	82.3
			11898	L	Missense	G	T	23733	82.3
			15167	L	Missense	G	A	29692	83.7
	PIV2_Fukushima_O861_2019 (LC720869)	HBTEC-ALI	9	-	SNV	T	C	3343	10.0
			4593	M	Silent	T	C	9818	30.1
			5163	F	Missense	C	T	15146	30.2
		Vero/TMPRSS2	3832	M	Missense	G	A	1767	52.9
			7341	HN	Missense	A	G	1363	14.0
			14518	L	Missense	G	T	2910	24.5
			15292	L	Silent	G	A	4417	20.5
			15444	L	Deletion	A	-	3764	10.5
		Vero	9	-	SNV	T	C	351	16.2
			2536	P	Missense	G	T	2733	17.3
			7404	HN	Missense	A	G	1860	94.1
			13615	L	Silent	T	C	3530	90.3
			14792	L	Missense	T	G	2355	27.6
Respirovirus pneumoniae	PIV3_Fukushima_O330_2018 (LC720876)	HBTEC-ALI	**27^28**	-	Insertion	-	T	311	11.6

(Human parainfluenzavirus 3)			**28**	-	SNV	A	T	326	13.8
			**6184**	F	Silent	T	C	11304	29.4
			**11351**	L	Silent	T	C	10246	10.5
		Vero/TMPRSS2	**6184**	F	Silent	T	C	9565	99.9
			8471	HN	Missense	A	G	2794	99.8
	PIV3_Fukushima_O644_2019 (LC720877)	HBTEC-ALI	**28**	-	SNV	A	T	872	10.1
			7383	HN	Missense	C	T	32045	31.7
		Vero/TMPRSS2	1303	N	Missense	G	T	10347	42.3
			2420	P	Missense	G	A	6423	70.8
			5242	F	Missense	A	G	2900	68.4
			7379	HN	Missense	A	G	5216	22.2
			7493	HN	Missense	A	G	5932	71.1
			8687	L	Silent	T	C	3246	23.4
	PIV3_Fukushima_O726_2019 (LC720879)	HBTEC-ALI	2090	P	Missense	G	C	8670	52.9
			**6900**	HN	Missense	T	C	14088	10.1
			12916	L	Missense	T	C	6180	11.2
		Vero/TMPRSS2	**6900**	HN	Missense	T	C	6565	99.8
			**6964**	HN	Silent	T	C	9321	99.8
			7379	HN	Missense	A	G	6783	98.5
			7451	HN	Missense	G	A	6057	99.2
Orthorubulavirus hominis	PIV4a_Fukushima_H725_2019 (LC706553)	HBTEC-ALI	**5821**	F	Silent	C	T	6797	32.5
(Human parainfluenzavirus 4)			**9237**	HN	Missense	A	G	10019	53.5
			**16235^16236**	L	Insertion	-	T	2645	10.1
		Vero/TMPRSS2	**4110**	M	Missense	A	G	1434	99.4
			**5821**	F	Silent	C	T	679	99.7
			8049	HN	Missense	A	T	733	84.3
			**9237**	HN	Missense	A	G	1136	98.9
			16263	L	Missense	A	T	604	14.2
	PIV4b_Fukushima_O896_2019 (LC706554)	HBTEC-ALI	3736	M	Silent	G	A	11956	27.7
			**6450**	F	Silent	T	C	7452	99.9
			**7536**	-	SNV	A	G	4173	99.9
			9232	HN	Missense	G	A	5024	24.1
			17353^17354	-	Insertion	-	CTTGGT	125	39.2
		Vero/TMPRSS2	**758**	N	Silent	G	A	1247	81.5
			**3454**	-	SNV	A	G	3630	81.1
			**4276**	M	Silent	C	T	1306	84.0
			4430	M	Missense	G	A	2052	19.8
			**6450**	F	Silent	T	C	1465	18.2
			**7536**	-	SNV	A	G	738	17.6
			8555	HN	Missense	C	T	1276	61.3
			9031	HN	Missense	A	G	1754	12.8
			**13865**	L	Silent	A	G	2418	81.3
			17353^17354	-	Insertion	-	CTTG	25	88.0
	PIV4b_Fukushima_OR476_2022 (LC706556)	HBTEC-ALI	8154	HN	Missense	G	A	11713	36.8
			**8561**	HN	Silent	A	G	4840	40.0
			9028	HN	Missense	C	T	9747	28.0
			**9119**	HN	Silent	C	T	9813	39.9
		Vero/TMPRSS2	**2419**	P	Missense	A	T	223	100.0
			2549^2550	P	Insertion *	-	GG	187	10.7
			**8152**	HN	Missense	G	A	188	99.5
			9022	HN	Missense	C	A	210	79.5
			10479	L	Missense	G	T	106	11.3
			14160	L	Missense	A	G	157	10.2
			16074	L	Missense	G	A	137	19.7
			17038	-	SNV	A	G	3016	24.0
			17083	-	SNV	A	G	5559	28.6
			17094	-	SNV	A	G	5646	29.2
			17099	-	SNV	A	G	5716	29.6
			17166	-	SNV	A	G	5147	33.8
			17212	-	SNV	A	G	2065	46.3
			17220..17221	-	MNV	AA	GG	1999	45.1
			17231	-	SNV	A	G	1776	41.6
			17242	-	SNV	A	G	1399	32.0

N; nucleocapsid protein, P; phosphoprotein, M; matrix protein, F; fusion glycoprotein, HN; hemagglutinin-neuraminidase glycoprotein, L; large protein.

* RNA editing site, **Bold**; quasispecies present in both P0 and P5.

Table S5. List of the quasispecies that originally existed in the P0 inoculum at >1% frequency, including location, coverage and frequency

Species	Name (Genbank ID)	Culture (Isolate)	Region	Gene	Type *	Reference	Allele	Coverage	Frequency
Respirovirus laryngotracheitidis	PIV1_Fukushima_O77_2018 (LC764863)	HBTEC-ALI	2596	P	Silent	C	T	27442	2.5
(Human parainfluenzavirus 1)			3734^3735	M	Insertion	-	A	8508	2.7
			4097	M	Silent	C	T	7582	1.6
			4520^4521	M	Insertion	-	A	14386	4.0
			4970^4971	-	Insertion	-	A	4560	1.4
			5408	F	Silent	C	T	9904	2.9
			5745^5746	F	Insertion	-	A	5300	2.0
			**8840**	L	Silent	T	C	4066	19.7
			9090^9091	L	Insertion	-	T	4357	2.0
			9379^9380	L	Insertion	-	T	2941	1.2
			9830^9831	L	Insertion	-	T	6603	3.7
			9908^9909	L	Insertion	-	A	4979	1.3
			10661^10662	L	Insertion	-	A	4542	1.2
			10726^10727	L	Insertion	-	T	5676	3.4
			10745^10746	L	Insertion	-	A	5638	4.0
			11124^11125	L	Insertion	-	A	9551	4.1
			12177^12178	L	Insertion	-	A	11509	1.1
			13693^13694	L	Insertion	-	T	6559	3.5
			13987^13988	L	Insertion	-	A	4800	1.4
	PIV1_Fukushima_H171_2018 (LC764862)	HBTEC-ALI	7	-	SNV	A	T	208	1.4
			**3012**	P	Missense	A	G	3019	19.5
			3731^3732	M	Insertion	-	A	5799	2.7
			4872^4873	-	Insertion	-	A	7438	1.1
			4967^4968	-	Insertion	-	A	5225	1.8
			5742^5743	F	Insertion	-	A	4216	2.0
			**8277**	HN	Missense	T	C	7580	2.5
			8990	L	Silent	C	T	3855	1.1
			9087^9088	L	Insertion	-	T	3008	2.0
			9376^9377	L	Insertion	-	T	3397	1.2
			9827^9828	L	Insertion	-	T	6291	3.1
			9905^9906	L	Insertion	-	A	3374	1.5
			10723^10724	L	Insertion	-	T	4025	1.7
			10742^10743	L	Insertion	-	A	4080	3.7
			11121^11122	L	Insertion	-	A	6806	4.4
			12174^12175	L	Insertion	-	A	11450	1.2
			13984^13985	L	Insertion	-	A	4161	1.2
			14956^14957	L	Insertion	-	A	1664	1.3
			15524^15525	-	Insertion	-	A	1749	1.0
	PIV1_Fukushima_O425_2018 (LC764864)	HBTEC-ALI	**4**	-	SNV	G	A	121	2.5
			5	-	SNV	A	T	125	1.6
			3404	P	Silent	T	C	5525	1.4
			4870^4871	-	Insertion	-	A	7819	1.2
			5063	-	SNV	A	G	6208	1.5
			**7377**	HN	Silent	C	T	2644	6.2
			**8448**	HN	Missense	T	A	5551	1.0
			9085^9086	L	Insertion	-	T	3390	2.2
			9374^9375	L	Insertion	-	T	3516	1.4
			9825^9826	L	Insertion	-	T	4268	2.1
			9903^9904	L	Insertion	-	A	3022	1.2
			10740^10741	L	Insertion	-	A	3553	2.7
			11119^11120	L	Insertion	-	A	6049	5.1
			12172^12173	L	Insertion	-	A	8861	1.1
			13688^13689	L	Insertion	-	T	2455	1.7
	PIV1_Fukushima_O503_2019 (LC764865)	HBTEC-ALI	12	-	Deletion	A	-	181	1.1
			3363	P	Missense	G	A	11994	1.0
			4515^4516	M	Insertion	-	A	9474	3.8
			4870^4871	-	Insertion	-	A	8251	1.5
			**5492**	F	Missense	C	T	7203	27.5
			5811^5812	F	Insertion	-	A	2913	1.0
			6887	HN	Missense	A	G	8947	1.8
			8713	-	SNV	A	G	2161	2.9
			9085^9086	L	Insertion	-	T	3210	2.7
			9374^9375	L	Insertion	-	T	3339	1.0
			9825^9826	L	Insertion	-	T	4275	2.3
			10740^10741	L	Insertion	-	A	3623	2.9
			11119^11120	L	Insertion	-	A	5992	5.2
			13688^13689	L	Insertion	-	T	2602	2.7
			13982^13983	L	Insertion	-	A	3453	1.3
Orthorubulavirus laryngotracheitidis	PIV2_Fukushima_O13_2018 (LC720864)	HBTEC-ALI	1915^1916	-	Insertion	-	A	94448	2.8
(Human parainfluenzavirus 2)			3478	-	SNV	A	G	74028	1.1
			4738^4739	-	Insertion	-	A	72467	2.5
			6627^6628	-	Insertion	-	A	98817	1.7
			8740^8741	-	Insertion	-	A	51008	1.0
			10001^10002	L	Insertion	-	T	95113	1.3
			12045^12046	L	Insertion	-	T	78038	1.1

			14709^14710	L	Insertion	-	T	92305	4.6
	PIV2_Fukushima_O33_2018 (LC720865)	HBTEC-ALI	3361^3362	-	Insertion	-	A	66872	2.5
			4027	M	Silent	G	A	95442	5.8
			4057	M	Silent	G	A	94052	6.3
			4738^4739	-	Insertion	-	A	72060	2.1
			10001^10002	L	Insertion	-	T	78598	2.1
			12045^12046	L	Insertion	-	T	62884	1.3
			14709^14710	L	Insertion	-	T	83550	6.4
	PIV2_Fukushima_O836_2019 (LC720868)	HBTEC-ALI	1906^1907	-	Insertion	-	A	77058	2.6
			3352^3353	-	Insertion	-	A	71614	2.9
			4447	M	Silent	G	A	76786	2.0
			4729^4730	-	Insertion	-	A	69846	2.4
			6618^6619	-	Insertion	-	A	75218	1.8
			**8441**	HN	Missense	T	A	52223	3.4
			8731^8732	-	Insertion	-	A	30453	1.0
			9992^9993	L	Insertion	-	T	78521	1.4
			12036^12037	L	Insertion	-	T	57734	1.2
			12392	L	Missense	T	A	90046	1.2
			13294	L	Missense	C	T	67160	1.4
			13665	L	Silent	T	C	93161	19.7
			14700^14701	L	Insertion	-	T	76795	4.9
	PIV2_Fukushima_O861_2019 (LC720869)	HBTEC-ALI	1910^1911	-	Insertion	-	A	81831	2.6
			3356^3357	-	Insertion	-	A	90985	2.6
			3365^3366	-	Insertion	-	A	90054	1.8
			3785	M	Silent	G	A	97879	3.1
			4733^4734	-	Insertion	-	A	80097	2.6
			5789^5790	F	Insertion	-	T	97412	1.5
			6622^6623	-	Insertion	-	A	93876	1.8
			9996^9997	L	Insertion	-	T	93221	1.4
			12040^12041	L	Insertion	-	T	70787	1.1
			14704^14705	L	Insertion	-	T	91656	4.7
Respirovirus pneumoniae	PIV3_Fukushima_O330_2018 (LC720876)	HBTEC-ALI	**27^28**	-	Insertion	-	T	542	1.1
(Human parainfluenzavirus 3)			**28**	-	SNV	A	T	559	2.5
			2497^2498	P	Insertion	-	A	18132	2.9
			3873	M	Missense	G	A	11374	1.1
			4944^4945	-	Insertion	-	A	16966	3.6
			**6184**	F	Silent	T	C	15163	14.8
			6723^6724	-	Insertion	-	A	5497	1.3
			6774^6775	-	Insertion	-	A	6015	1.8
			6869	HN	Missense	G	A	13239	1.3
			9721^9722	L	Insertion	-	T	6625	1.4
			10637^10638	L	Insertion	-	A	8089	1.0
			**11351**	L	Silent	T	C	12304	1.5
			11614^11615	L	Insertion	-	T	13578	1.7
			12069^12070	L	Insertion	-	A	12822	1.1
			13038	L	SNV	G	A	9293	2.4
			15157^15158	L	Insertion	-	A	6975	1.2
			15175	L	SNV	A	G	6658	3.2
			15424^15425	-	Insertion	-	AT	3561	1.6
	PIV3_Fukushima_O644_2019 (LC720877)	HBTEC-ALI	27^28	-	Insertion	-	T	272	4.8
			**28**	-	SNV	A	T	273	3.7
			2497^2498	P	Insertion	-	A	10855	3.0
			3427^3428	P	Insertion	-	A	5493	1.0
			4944^4945	-	Insertion	-	A	6839	2.3
			6723^6724	-	Insertion	-	A	2500	1.6
			9721^9722	L	Insertion	-	T	2965	2.0
			10637^10638	L	Insertion	-	A	4040	1.2
			11614^11615	L	Insertion	-	T	6727	1.7
			12069^12070	L	Insertion	-	A	6371	1.3
			12710	L	Silent	C	T	3621	2.0
			15157^15158	L	Insertion	-	A	3252	1.6
	PIV3_Fukushima_O726_2019 (LC720879)	HBTEC-ALI	27^28	-	Insertion	-	T	158	1.9
			28	-	SNV	A	T	160	2.5
			2497^2498	P	Insertion	-	A	4646	3.3
			2652	P	Missense	A	G	8659	3.4
			3427^3428	P	Insertion	-	A	2723	1.4
			4944^4945	-	Insertion	-	A	3700	2.9
			5052	-	SNV	C	T	3341	1.5
			6723^6724	-	Insertion	-	A	1393	1.5
			**6900**	HN	Missense	T	C	2748	6.1
			**6964**	HN	Silent	T	C	3177	5.3
			7331	HN	Missense	C	T	3003	4.9
			10517	L	Silent	T	C	1712	1.7
			14810	L	Silent	C	T	2128	2.8

			15157^15158	L	Insertion	-	A	1834	1.3
			15424^15425	-	Insertion	-	AT	853	1.8
Orthorubulavirus hominis (Human parainfluenzavirus 4)	PIV4a_Fukushima_H725_2019 (LC706553)	HBTEC-ALI	9	-	Deletion	A	-	1816	2.7
			30^31	-	Insertion	-	A	6067	1.5
			932^933	N	Insertion	-	AT	15034	1.3
			1854^1855	-	Insertion	-	A	19703	2.4
			2008^2009	-	Insertion	-	A	9606	1.2
			2465^2466	P	Insertion	-	A	8922	3.1
			2500^2501	P	Insertion	-	A	10596	2.0
			3542^3543	-	Insertion	-	A	14136	5.7
			3692^3693	M	Insertion	-	A	8809	1.5
			**4110**	M	Missense	A	G	14273	1.3
			4657^4658	M	Insertion	-	A	17216	2.5
			5105^5106	-	Insertion	-	A	14484	2.7
			5491	F	Silent	T	C	8218	1.7
			5497	F	Silent	T	C	8170	1.8
			5656^5657	F	Insertion	-	A	7514	4.5
			**5821**	F	Silent	C	T	9096	15.2
			6889	-	SNV	C	T	22006	1.2
			6939	-	SNV	C	T	21380	2.4
			7041^7042	-	Insertion	-	A	23095	5.8
			7052	-	SNV	A	G	26241	1.3
			7197	-	SNV	C	G	23030	1.5
			7276^7277	-	Insertion	-	A	20924	1.7
			7555^7556	-	Insertion	-	A	11292	8.2
			8337^8338	HN	Insertion	-	A	14582	2.5
			8574^8575	HN	Insertion	-	T	10926	1.1
			8581	HN	SNV	C	T	10577	1.7
			**9237**	HN	Missense	A	G	10754	35.2
			9284	HN	Missense	C	T	11068	3.3
			9819^9820	-	Insertion	-	A	7865	1.5
			9958^9959	-	Insertion	-	A	11857	1.1
			10320^10321	L	Insertion	-	T	8518	1.4
			10421^10422	L	Insertion	-	T	10277	1.3
			11075^11076	L	Insertion	-	T	13719	1.9
			11238^11239	L	Insertion	-	T	10227	1.2
			12592^12593	L	Insertion	-	T	9940	2.1
			15894^15895	L	Insertion	-	A	11571	3.1
			**16235^16236**	L	Insertion	-	T	9093	8.2
			16745^16746	L	Insertion	-	T	8652	3.8
			16960^16961	-	Insertion	-	A	4125	1.8
			17039^17040	-	Insertion	-	A	3854	2.4
	PIV4b_Fukushima_O896_2019 (LC706554)	HBTEC-ALI	**758**	N	Silent	G	A	157	3.2
			1603	N	Missense	A	G	243	1.6
			1691	N	Missense	T	C	258	7.0
			2496^2497	P	Insertion	-	A	115	2.6
			2547^2548	P	Insertion *	-	G	130	1.5
			2547^2548	P	Insertion *	-	GG	130	3.1
			2556	P	Missense	T	C	166	13.9
			2601..2602	P	Deletion	AA	-	188	1.1
			2870^2871	P	Insertion	-	A	101	4.0
			3003	P	Silent	T	C	100	2.0
			3313	-	SNV	G	T	249	4.4
			3423	-	SNV	A	G	201	1.5
			**3454**	-	SNV	A	G	190	41.1
			3688^3689	M	Insertion	-	A	121	5.8
			**4276**	M	Silent	C	T	116	2.6
			5040	-	SNV	A	G	295	5.1
			5101^5102	-	Insertion	-	A	240	2.1
			5550	F	Silent	A	G	148	1.4
			5652^5653	F	Insertion	-	A	157	1.9
			**6450**	F	Silent	T	C	94	19.1
			6999	-	SNV	A	G	361	1.1
			7044^7045	-	Insertion	-	A	353	2.3
			7266^7267	-	Insertion	-	A	140	3.6
			7267	-	Deletion	A	-	141	1.4
			**7536**	-	SNV	A	G	55	18.2
			8339	HN	Missense	C	G	108	1.9
			9025	HN	Missense	C	T	125	9.6

		9030^9031	HN	Insertion	-	A	122	1.6
		9452	-	SNV	A	G	119	2.5
		9809^9810	-	Insertion	-	A	134	3.0
		10411^10412	L	Insertion	-	T	69	2.9
		12095	L	Silent	C	T	88	2.3
		12284	L	Silent	A	G	72	2.8
		12559^12560	L	Insertion	-	T	93	2.2
		12582^12583	L	Insertion	-	T	87	2.3
		**13865**	L	Silent	A	G	113	37.2
		14232	L	Missense	T	C	72	2.8
		16813	L	Missense	A	G	134	1.5
PIV4b_Fukushima_OR476_2022 (LC706556)	HBTEC-ALI	7	-	SNV	A	G	716	1.3
		**2419**	P	Missense	A	T	2227	2.2
		2498^2499	P	Insertion	-	A	2010	2.1
		2872^2873	P	Insertion	-	A	2123	2.0
		3367	-	SNV	C	T	7672	2.0
		3540^3541	-	Insertion	-	A	2407	3.4
		4655^4656	M	Insertion	-	A	2806	3.2
		4801	-	SNV	C	T	5758	1.2
		5654^5655	F	Insertion	-	A	1844	4.6
		7196	-	SNV	C	T	5243	1.1
		7268^7269	-	Insertion	-	A	2842	3.8
		7321	-	SNV	C	T	3444	1.7
		**8152**	HN	Missense	G	A	2762	2.5
		**8561**	HN	Silent	A	G	1241	37.4
		8566^8567	HN	Insertion	-	T	1181	1.0
		8573	HN	Silent	C	T	1158	1.5
		8775	HN	Missense	C	A	1799	1.4
		**9119**	HN	Silent	C	T	1785	36.5
		10273	L	Silent	A	G	1835	3.4
		10312^10313	L	Insertion	-	T	2020	2.2
		10413^10414	L	Insertion	-	T	1380	1.6
		10618^10619	L	Insertion	-	A	1397	1.1
		12561^12562	L	Insertion	-	T	1506	2.5
		12562	L	Deletion	T	-	1544	1.2
		12584^12585	L	Insertion	-	T	1683	1.8
		15886^15887	L	Insertion	-	A	1863	3.1
		16952^16953	-	Insertion	-	A	1512	1.5

N; nucleocapsid protein, P; phosphoprotein, M; matrix protein, F; fusion glycoprotein, HN; hemagglutinin-neuraminidase glycoprotein, L; large protein.

* RNA editing site, **Bold**; quasispecies present in both P0 and P5.

Table S6. Residues of the clevage site in fusion proteins

Isolate	P7	P6	P5	P4	P3	P2	P1	P'1	P'2	P'3
HPIV1	A/N	D	N/V	P	Q	S/T	R	F	F	G
PIV1_Fukushima_O77_2018 (LC764863)	N	D	N	P	Q	T	R	F	F	G
PIV1_Fukushima_H171_2018 (LC764862)	N	D	N	P	Q	T	R	F	F	G
PIV1_Fukushima_O425_2018 (LC764864)	N	D	N	P	Q	T	R	F	F	G
PIV1_Fukushima_O503_2019 (LC764865)	N	D	N	P	Q	T	R	F	F	G
HPIV2 (trypsin dependent)	T	A/K/T	P/T	R	Q	E	R	F	A	G
HPIV2 (trypsin independent)	T	K/T	T	R	Q	K	R	F	A	G
PIV2_Fukushima_O13_2018 (LC720864)	T	K	P	R	R	E	R	F	A	G
PIV2_Fukushima_O33_2018 (LC720865)	T	K	P	R	R	E	R	F	A	G
PIV2_Fukushima_O836_2019 (LC720868)	T	K	P	R	R	E	R	F	A	G
PIV2_Fukushima_O861_2019 (LC720869)	T	K	P	R	R	E	R	F	A	G
HPIV3 (trypsin dependent)	T	D	P	R	T	E	R	F	F	G
HPIV3 (trypsin independent)	T	D/N	P	R/T	T	K/R	R	F	F	G
PIV3_Fukushima_O330_2018 (LC720876)	T	D	P	R	T	E	R	F	F	G
PIV3_Fukushima_O644_2019 (LC720877)	T	D	P	R	T	E	R	F	F	G
PIV3_Fukushima_O726_2019 (LC720879)	T	D	P	R	T	E	R	F	F	G
HPIV4a	S	S	E	I	Q	S	R	F	F	G
PIV4a_Fukushima_H725_2019 (LC706553)	S	S	E	V	Q	S	R	F	F	G
HPIV4b	S	S	E	I	Q	S	R	F	F	G
PIV4b_Fukushima_O896_2019 (LC706554)	S	S	E	I	Q	S	R	F	F	G
PIV4b_Fukushima_OR476_2022 (LC706556)	S	S	E	I	Q	S	R	F	F	G

Residues from previous reports are shown, and in gray if they are identical to those of the isolates used in this study. (Abe M, Tahara M, Sakai K, Yamaguchi H, Kanou K, Shirato K, Kawase M, Noda M, Kimura H, Matsuyama S, Fukuhara H, Mizuta K, Maenaka K, Ami Y, Esumi M, Kato A, Takeda M. 2013. TMPRSS2 is an activating protease for respiratory parainfluenza viruses. J Virol 87:11930-5.)
